# Homology modeling, virtual screening, molecular docking, and dynamics studies for discovering *Staphylococcus epidermidis* FtsZ inhibitors

**DOI:** 10.3389/fmolb.2023.1087676

**Published:** 2023-03-03

**Authors:** Divya Vemula, Dhanashri Ramesh Maddi, Vasundhra Bhandari

**Affiliations:** National Institute of Pharmaceutical Education and Research, Hyderabad, India

**Keywords:** FtsZ, *Staphylococcus* epidermidis, insilico analysis, molecular modeling and simulation, virtual screeening

## Abstract

*Staphylococcus epidermidis* is the most common cause of medical device-associated infections and is an opportunistic biofilm former. Among hospitalized patients, *S. epidermidis* infections are the most prevalent, and resistant to most antibiotics. In order to overcome this resistance, it is imperative to treat the infection at a cellular level. The present study aims to identify inhibitors of the prokaryotic cell division protein FtsZ a widely conserved component of bacterial cytokinesis. Two substrate binding sites are present on the FtsZ protein; the nucleotide-binding domain and the inter-domain binding sites. Molecular modeling was used to identify potential inhibitors against the binding sites of the FtsZ protein. One hundred thirty-eight chemical entities were virtually screened for the binding sites and revealed ten molecules, each with good binding affinities (docking score range −9.549 to −4.290 kcal/mol) compared to the reference control drug, i.e., Dacomitinib (−4.450 kcal/mol) and PC190723 (−4.694 kcal/mol) at nucleotide and inter-domain binding sites respectively. These top 10 hits were further analyzed for their ADMET properties and molecular dynamics simulations. The Chloro-derivative of GTP, naphthalene-1,3-diyl bis(3,4,5-trihydroxybenzoate), Guanosine triphosphate (GTP), morpholine and methylpiperazine derivative of GTP were identified as the lead molecules for nucleotide binding site whereas for inter-domain binding site, 1-(((amino(iminio)methyl)amino)methyl)-3-(3-(tert-butyl)phenyl)-6,7-dimethoxyisoquinolin-2-ium, and Chlorogenic acidwere identified as lead molecules. Molecular dynamics simulation and post MM/GBSA analysis of the complexes revealed good protein-ligand stability predicting them as potential inhibitors of FtsZ (Figure 1). Thus, identified FtsZ inhibitors are a promising lead compounds for *S. epidermidis* related infections.

## Introduction

Antibiotic resistance is a global issue associated with high morbidity and mortality (Akova, 2016). Multidrug-resistant bacteria and significant bacterial infections exhibit alarming rates of emergence and resistance to standard antibiotics. Currently, there are no viable preventative measures or effective medicines, and only a limited number of new antibiotics are developing, making the fight against bacterial infections even more challenging. Innovation is necessary until new treatment alternatives and antimicrobial therapies are developed (Chellat et al., 2016). Focusing on new targets or crucial mechanisms for identifying potential treatment is essential. We focused on cell division, a fundamental and vital process. Binary fission is a standard process in bacteria to produce offspring. Filamenting temperature-sensitive mutant Z (FtsZ) acts as a pacemaker for the formation of divisomes (macromolecular protein complexes that mediate the distinct and unique phases of bacterial cell division) during cytokinesis by assembling protofilaments to form the FtsZ-ring (also known as the Z-ring) at the site of potential division (Silber et al., 2020).

*Staphylococcus epidermidis*, belonging to the staphylococci family, has been identified as a significant contributor of nosocomial infection and recognized as an important opportunistic pathogen. (Widerström, 2016). Currently, its rate of nosocomial infections is on par with that of *Staphylococcus aureus*, one of its more dangerous kin (National Nosocomial Infections Surveillance System, 2004). *S. epidermidis* and other coagulase-negative staphylococci were mainly found responsible for causing medical device-associated infections (Kleinschmidt et al., 2015). These species are highly contagious among prosthetic valves, cardiac devices, central lines, catheters, and IV drug use patients. In addition, neonates are found to be highly susceptible to them (Cheung and Otto, 2010). Approximately 20%–30% of orthopaedic device-related infections (ODRIs) (Trampuz and Zimmerli, 2006; Montanaro et al., 2011; Moriarty et al., 2016) are caused by *S. epidermidis*, and in late-developing infections, the incidence may potentially reach 50% (Schafer et al., 2008). When studying the clinical course and outcome of staphylococcal ODRIs in older patients, Morgenstern et al. were able to demonstrate that *S. epidermidis* was linked to extended infections and had a lower cure rate (75%) than *S. aureus* (84%) (Morgenstern et al., 2016).

FtsZ is a crucial component of the cytoskeletal protein complex in bacterial cytokinesis (Erickson et al., 2010). Current technologies in the discovery of antibiotics have identified compounds that directly interact with the crucial cell division protein FtsZ, disturbing the dynamics and operation of the cell division machinery, or degrading FtsZ, damaging the structural integrity (Silber et al., 2020). As a result of prokaryotes’ significant protein conservation, FtsZ is present in various pathogens, such as *Escherichia coli, Staphylococcus aureus, Mycobacterium tuberculosis, Mycoplasma genitalium, Mycoplasma pneumoniae, Helicobacter pylori, Treponema pallidum, Neisseria meningitidis, Rickettsia prowazekii, Campylobacter jejuni, Shigella, and Salmonella* (Chalker, A.F. and Lunsford, R.D., 2002).

FtsZ mainly consists of two binding sites: a nucleotide-binding site and an inter-domain binding site (Casiraghi et al., 2020) which includes a C-terminal tail (CTT) and C-terminal variable region (CTV) connected by a central helix (Oliva et al., 2007). In the nucleotide-binding site, GTP hydrolysis causes the protofilament to break down, weakening the protein-nucleotide connection, and ultimately preventing cell division. As the nucleotide-binding site is highly conserved among wide range of bacterial species, it became a crucial target for developing broad-spectrum antibacterial agents (Du and Lutkenhaus, 2019). Another functional site of FtsZ, located in a substantial cleft between the C-terminal domain and the H7 helix, is the inter-domain binding site. Various bacterial species have different cleft sizes, amino acid residue counts, and conservation rates. For instance, the inter-domain cleft is less conserved in Gram-negative bacteria than in Gram-positive bacteria. In accordance with the H7 helix’s curvature, the size of the interdomain cleft differs between bacterial species. FtsZ’s enzymatic domain has been shown to function as a self-activating GTPase (De Boer et al., 1992; RayChaudhuri, D. and Park, J.T., 1992). Similar to *S. aureus*, the FtsZ of *S. epidermidis* comprises two globular subdomains, the N- and C-terminal subdomains, which are connected by a synergy loop (T7 loop) and the H7 helix, which forms the centre of the structure. The N-terminal subdomain (residues 13–173) has a nucleotide-binding pocket i.e. nucleotide binding domain. Most likely, the C-terminal subdomain (residues 223–310) acts as a GTPase activating subdomain i.e., inter-domain binding site (Matsui et al., 2014). So, targeting an Inter-domain binding site of FtsZ can help design or develop target-specific drugs. Hence, we have screened compounds against the nucleotide and an inter-domain binding site of the FtsZ utilising molecular modeling methods.

## Materials and methods

**FIGURE 1:**
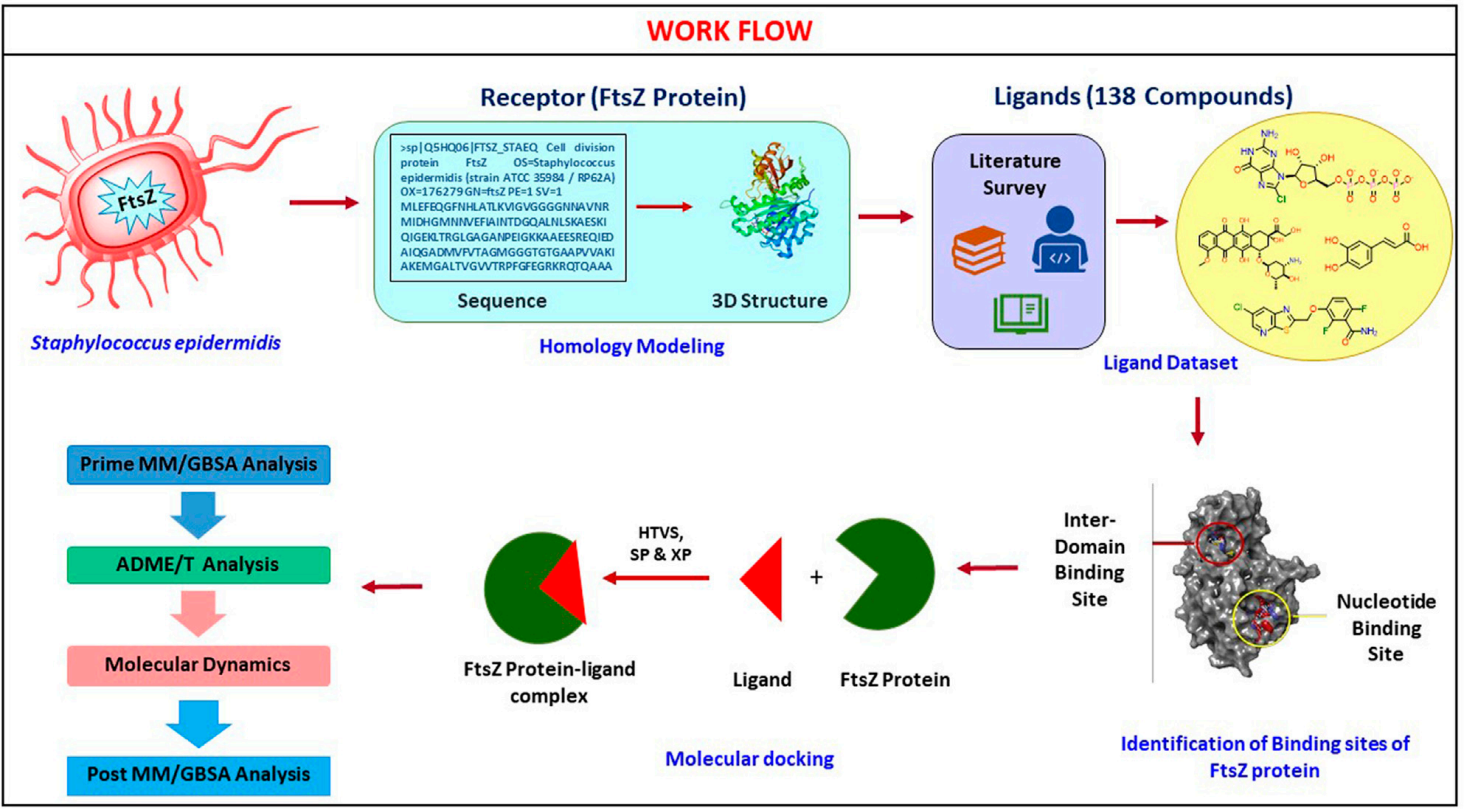
Computational screening workflow for identifying FtsZ inhibitors in *S. epidermidis.*

**FIGURE 2:**
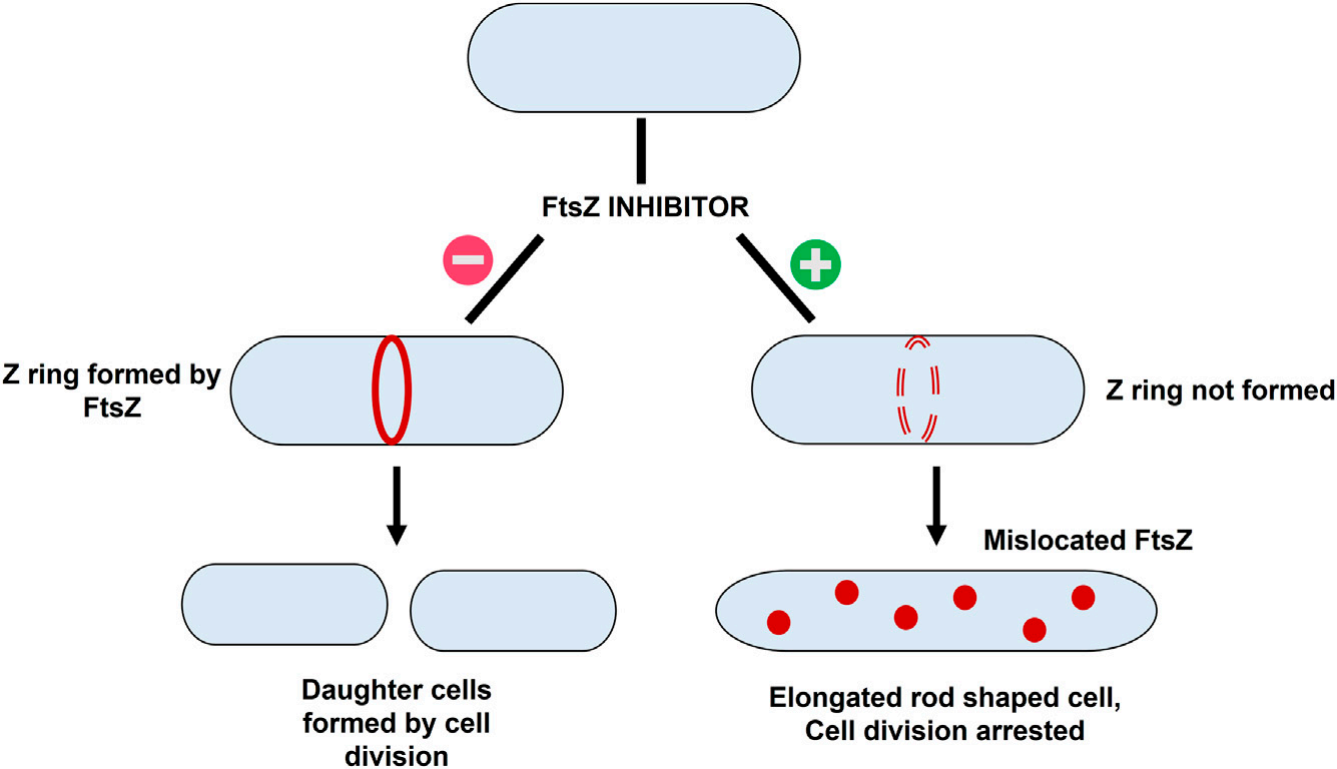
The mechanism of action of FtsZ inhibitors - i) Formation of Z ring in absence of FtsZ inhibitor resulting in bacterial cell division; ii) mislocation of FtsZ in presence of inhibitor resulting in elongation of filaments into rod shaped cells thereby causing cell division arrest.

The basic workflow of finding potential FtsZ inhibitors in *S. epidermidis* was discussed in Figure 1 and the mechanism of action of FtsZ inhibitor was explained in Figure 2.

### Conservation of FtsZ

The conservation of FtsZ protein was experimentally determined by performing BLAST analysis of whole UNIPROT database sequences. The default parameters like E-Threshold as 10, and the Auto-BLOSSUM 62 matrix were used. A pairwise sequence alignment of the two FtsZ sequences from *S. epidermidis* and *S. aureus* was performed using Clustal omega to identify the regions of similarity which indicates the structural, functional, and evolutionary relationship between the two sequences.

### Target preparation

The 3D structure of the *S. epidermidis* FtsZ protein was predicted using the Swiss-Model server because the detailed structural information of the crystallized structure is unavailable in PDB as the literature report of its PDB entry (4M8I) is not published. The protein sequence was retrieved from the Uniprot database (Uniprot ID: Q5HQ06). The template was selected based on the parameters observed in the BLAST findings by mainly focusing on the sequence similarity, resolution, and experimental technique used to determine the structure. (Choudhary et al., 2020). The target protein’s predicted 3D structure was validated using the Ramachandran (RC) Plot. The 3D protein structure was visualized using Maestro 13.1. After that, the built model was pre-processed using the protein preparation wizard of Schrodinger-suite 2022, which refine the protein structure for docking by setting bond orders, filling in missing loops, adding hydrogens, and deleting water molecules that are more than 3Å distances from the protein (Sahayarayan et al., 2021). After the H-bond assignment, H-bonds were optimized using PROPKA tool. The binding sites of the FtsZ protein were anticipated using the Site Map tool of the Schrodinger-suite 2022 which predicted best five sites for a given protein entry.

The receptor grid was generated using the “Receptor grid generation” panel from the glide module of Schrodinger software by preserving the grid’s default settings and size. A receptor grid was generated for both the nucleotide and inter-domain binding sites.

### Ligand preparation

The literature review obtained about 138 reported natural, semi-synthetic, and synthetic FtsZ inhibitors of various other pathogenic bacterial species. The experimentally proved, 138 natural, semi-synthetic, and synthetic FtsZ inhibitors of various other pathogenic bacterial species were obtained (Tripathy and Sahu, 2019). Among these ligands, the chemical structures of a few were obtained from PubChem, and the remainder were sketched using maestro 13.1’s 2D Sketcher. To prepare ligands, these structures were loaded into Schrodinger Workspace. The Ligprep module of Schrodinger-suite 2022 was used to prepare the ligands by optimizing their geometrical features and generating ionization states for the compounds to achieve the necessary pH of 7.0 ± 2.0.

### Molecular docking

Using the “ligand docking” panel of Schrodinger software, the prepared ligands were docked against both the nucleotide and inter-domain binding sites of the FtsZ protein. In order to generate hits from the ligand dataset, ligand docking was first carried out using the high throughput virtual screening (HTVS) approach with the precision mode set to HTVS, followed by standard precision (SP), and extra precision (XP). While taking the docking score into account, the Epik state penalties of the ligands were modified. (Kapusta et al., 2021). The docking validation is accomplished by redocking co-crystal ligands to their specific binding site of the receptor protein (El-Far et al., 2020). Dacomitinib (S2727), used as a control drug to assess the binding affinities and free energy of the proposed inhibitors, is a promising FtsZ inhibitor that Du et al. identified using *in vivo* and *in vitro* bioassays. while PC190723 (Elsen et al., 2012) served as the inter-domain binding site control drug. Instead of suppressing FtsZ filament assembly and condensation, PC190723 (difluoro-benzamide derivative) induces it (Andreu et al., 2010), causing FtsZ to assemble into delocalized cellular foci as opposed to the Z-ring (Adams et al., 2011).

### Prime MM/GBSA analysis

The binding free energies of the top 10 docked complexes (nucleotide-binding site and inter-domain binding site) were determined using the Prime MM/GBSA module of Schrodinger-suite (Muthumanickam et al., 2022; Ramachandran et al., 2022). The equation used for calculating free energy is as follows

$$\Delta G_{\mathrm{bind}}=G_{\mathrm{complex}}-(G_{\mathrm{protein}}+G_{\mathrm{ligand}})$$

The G_complex_ indicates complex energy, G_protein_ indicates receptor energy, and G_ligand_ indicates the unbound ligand energy.

### Prediction of ADME properties

The Qikprop module of Schrodinger’s suite 2022 was used to forecast the pharmacokinetic features, also known as absorption, distribution, metabolism, and excretion, of the top 10 hit compounds (Guo et al., 2016). This module provides information on the drug-like characteristics of the given ligands, such as molecular weight (mol MW), the number of hydrogen bond acceptors and donors (accptHB), solubility (QPlogS), Octanol/water Partition coefficient (QPlogPo/w), the percentage of oral absorption, apparent Caco-2 cell permeability (QPPCaco), and the brain/blood partition coefficient (QPlogBB).

### Toxicity prediction

ProTox—II was used to calculate the toxicity of the hit compounds. This programme delivers data based on chemical similarities, fingerprint propensities, etc. The ProTox-II employs machine learning models to determine the toxicity class, LD 50 values, organ toxicity, and toxicity endpoints, including hepatotoxicity, carcinogenicity, mutagenicity, immunogenicity, etc. Using a system that is universally accepted, toxicity classifications for chemicals are defined (GHS) (Banerjee et al., 2018). LD50 values are given in [mg/kg]:

Class I: If ingested, deadly (LD50 ≤ 5)
Class II: if ingested, deadly (5 < LD50 ≤ 50)
Class III: poisonous if ingested (50 < LD50 ≤ 300)
Class IV: Dangerous if ingested (300 < LD50 ≤ 2000)
Class V: May cause injury if ingested (2000 < LD50 ≤ 5000)
Class VI: Non-toxic (LD50 > 5000)

### Molecular dynamics (MD) simulation

The molecular dynamics (MD) simulation of the top docking scored protein-ligand complex was performed using the desmond module of Schrodinger suite 2022 for 100 ns. The simulation system was built using a system builder task where a simple point charge water (SPC) model was used as a solvent system, and periodic boundary condition (PBC) was applied using an orthorhombic boundary box. The system was neutralized by adding counter ions like Na^+^ and Cl^−^. The OPLS4 force field was used for energy minimization of molecular dynamics system. For the simulation of the build system it was loaded on to workspace, molecular dynamics simulation task was performed for 100 ns with a trajectory recording interval of 100 ps while the system was equilibrated with NPT ensemble system with pressure 1.01 bar and temperature 300 k, respectively. Finally, RMSD, RMSF, protein-ligand interaction and ligand properties were used for analyzing molecular dynamics simulation results (Kaushik et al., 2018).

### Post MM/GBSA analysis

Using the Prime MM/GBSA module of the Schrodinger, the post MM/GBSA analysis was carried out for the complexes at the 100 ns time frame of the molecular dynamics simulation to determine the binding free energies.

## Result and Discussion

### Conservation of FtsZ protein

**FIGURE 3:**
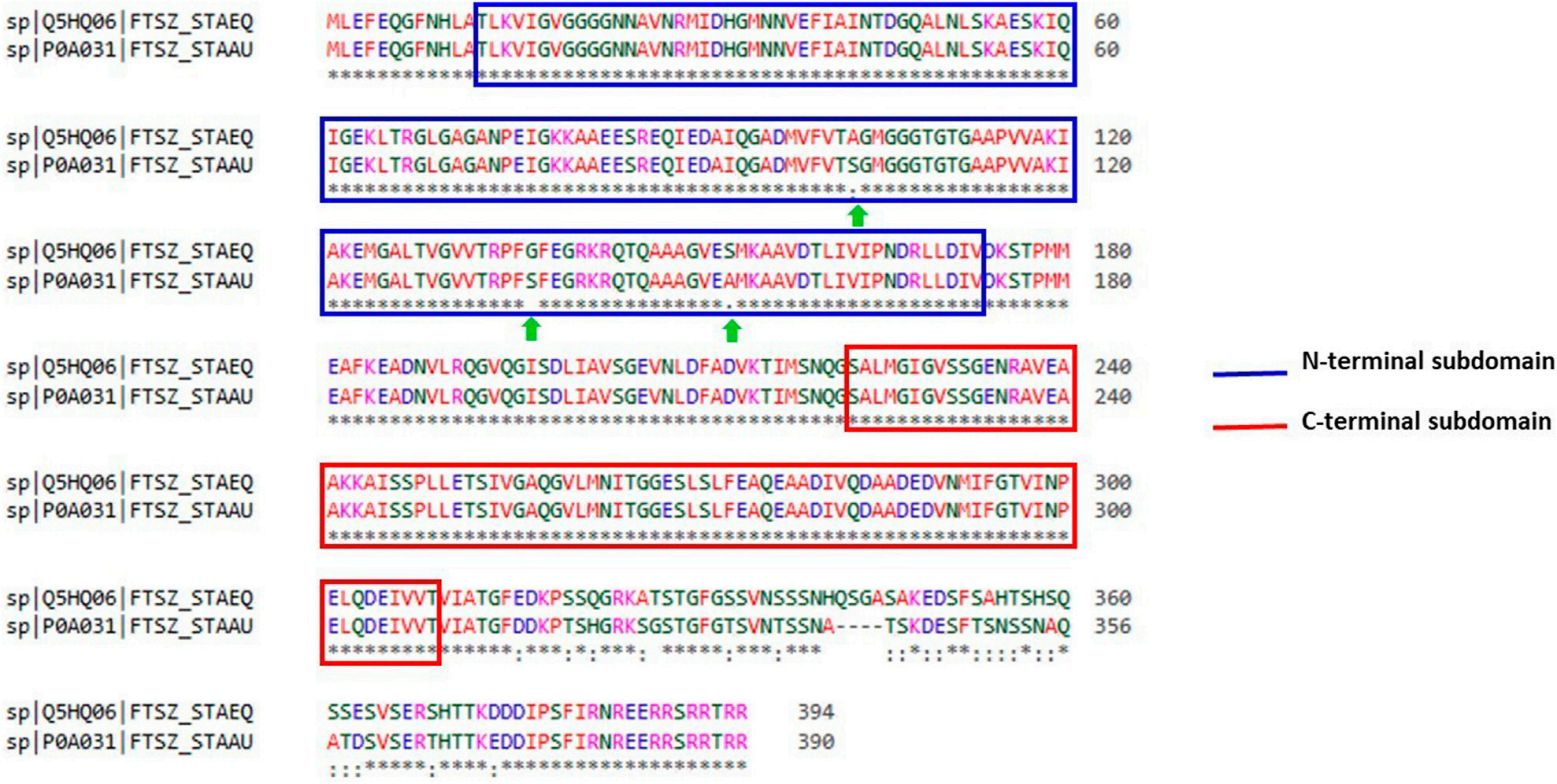
Sequence alignment of ftsz sequence (Uniprot ID: Q5HQ06 and P0A031) of *Staphylococcus epidermidis* and *Staphylococcus aureus*, respectively. Blue colour outlined box represents the N-terminal subdomain while Red colour outlined box indicates C-terminal subdomains of FtsZ protein with 100 percent similarity between these sequences.

With the aid of BLAST, the entire Uniprot database was aligned using the reference sequence of the FtsZ protein from S. epidermidis (Uniprot ID: Q5HQ06). This produced 250 hits, with the sequence identities ranging from 100% to 65.9%, indicating that they are the best matches or homologs present in different bacterial species such as *Staphylococcus species, Macrococcus caseolyticus, Abyssicoccus albus, Bacillus sp.* (Supplementary Figure S5) The FtsZ protein of *S. epidermidis* has shown a high sequence similarity about 92.39% identity with that of its kin i.e., *S. aureus* (Figure 3).

### Target preparation

**FIGURE 4:**
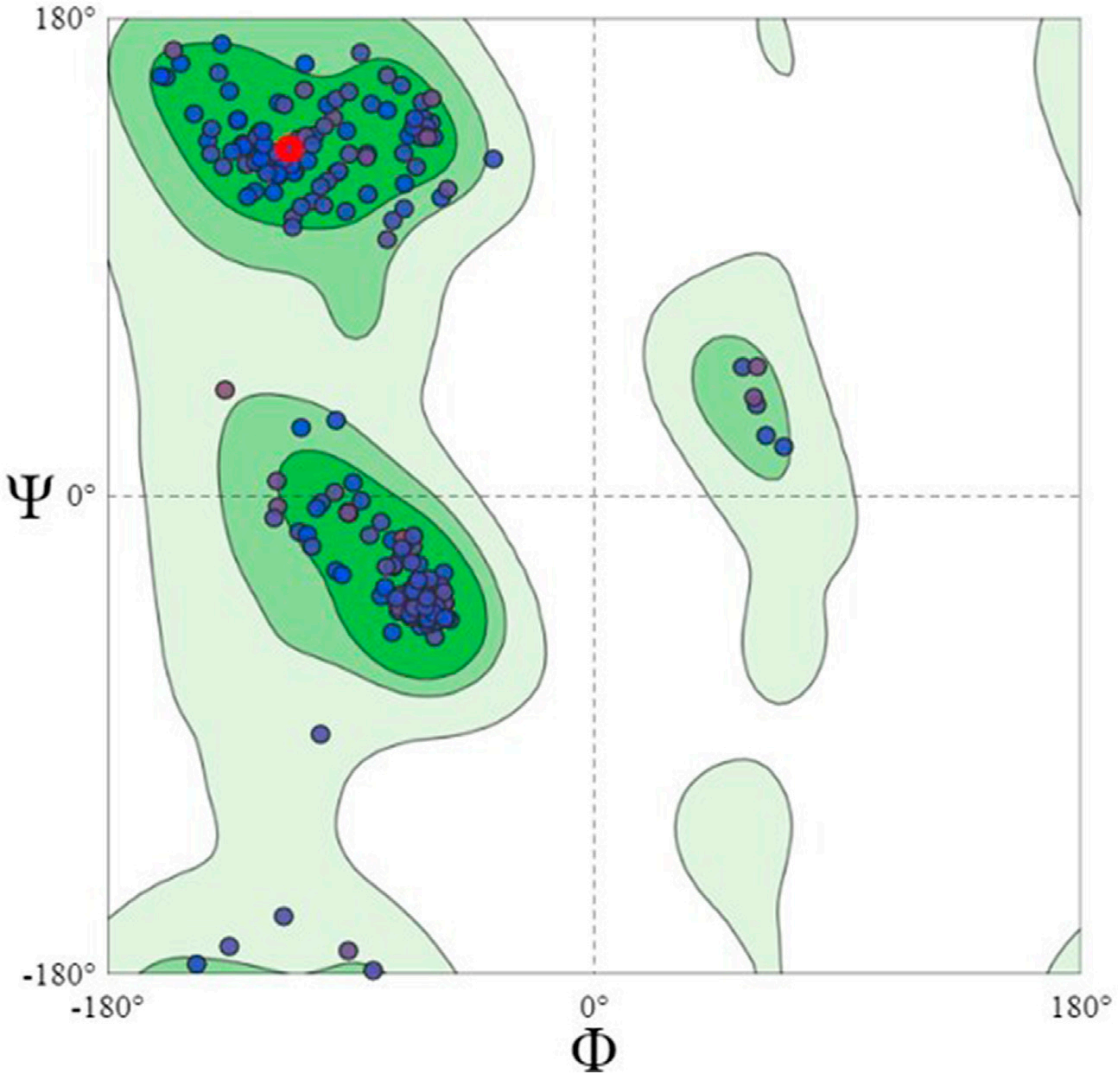
Ramachandran Plot of homology modeled FtsZ protein, dots indicate the amino acid residues of FtsZ protein. The residues present in dark green, light green and light grey regions of the plot represent allowed, favourable and disallowed regions of the plot, respectively. Most amino acids of the modeled protein are present in the allowed region of Ramachandran plot.

Using the Swiss model server, FtsZ protein homology modeling was carried out by choosing a template, PDB ID: 4M8I (Organism: *Staphylococcus epidermidis* RP62A) with 100% sequence identity and a Global Model Quality Estimation (GMQE) of 0.81%. The target protein’s 3D model was validated by the Ramachandran plot using the Swiss Model’s structure evaluation. (Figure 4). The 3D protein structure is visualized using Maestro 13.1). The active site for the nucleotide binding region was determined by using a co-crystallized ligand present in the template that we used for homology modeling followed by supplying the X, Y, and Z coordinates as -18.5, −9.81, and 19.98 Å, respectively, and retaining the other parameters at their default values, the receptor grid was generated at the nucleotide-binding site of FtsZ. The inter-domain binding site was predicted using the sitemap tool of Schrodinger-suite 2022. The top-ranked site showed a site score of interdomain binding site, volume = 116.620 Å^3^, hydrophilic score = 0.781, and hydrophobic score = 0.746. This predicted site contained Gln192, Gln195, Gly196, Asp199, Leu200, Val203, Leu209, Ile228, Leu261, Asn263, Ile264, Thr265, Val297, Asn299, Leu302, Val307, Thr309, and Ile311 residues. Similarly, the site coordinates X = −0.31, Y = 13.26, and Z = 24.61Å were provided to generate a receptor grid at the inter-domain binding site with the default settings for the remaining parameters.

### Molecular docking at nucleotide-binding site

Molecular docking was performed sequentially using the Schrodinger’s Glide module in three modes: HTVS, SP, and XP. It is crucial to know the binding affinity since docking was used to explore molecules that might inhibit the FtsZ protein. This research article discusses the outcomes of glide-XP docking since the extra precision (XP) mode generates precise results and uses the chemscore scoring program to evaluate the docked complex. The top 10 ligand structures are displayed in Table 1, along with their docking data and receptor-ligand interactions.

**TABLE 1:** Molecular docking results of top 10 hits at Nucleotide Binding Site of FtsZ.

SI No.	Ligand	Structure	Docking score (kcal/mol)	Interacting amino acids
1	Compound A	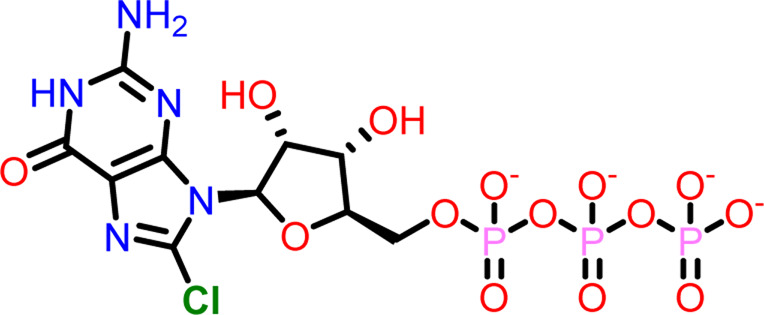	−9.549	Hydrogen Bond- Gly21, Gly22, Asn25, Arg29, Ala71, Ala73, Gly108, Thr109, Gly110, Thr133, Glu139, Asn166
		**((2R,3S,4R,5R)-5-(2-amino-8-chloro-6-oxo-1,6-dihydro-9H-purin-9-yl)-3,4-dihydroxytetrahydrofuran-2-yl)methyl triphosphate**		Pi-Pi Stacking- Phe183
2	Compound B	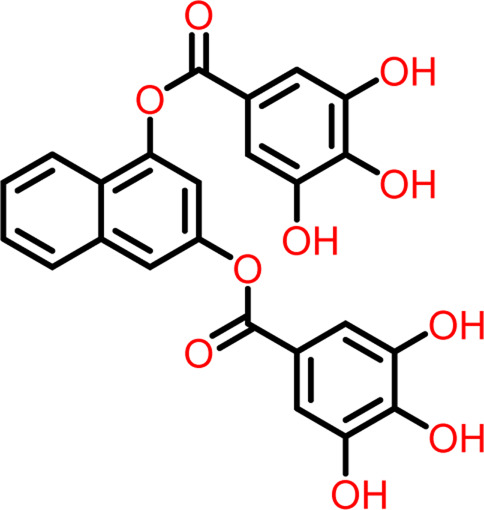	−9.539	Hydrogen Bond- Gly104, Gly108, Thr109, Gly110, Asn166, Asp187
		**naphthalene-1,3-diyl**		
		**bis(3,4,5-trihydroxybenzoate)**		Pi-Cation - Arg143
3	Compound C	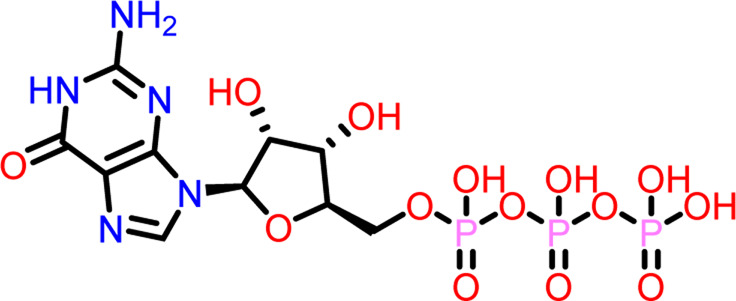	−9.530	Hydrogen Bond-
		**Guanosine-5′-triphosphate (GTP)**		Gly21, Gly22, Asn25, Arg29, Ala71, Gly108, Thr109, Thr133, Glu139, Arg143, Asn166
				Pi-Pi Stacking- Phe183
4	Compound D	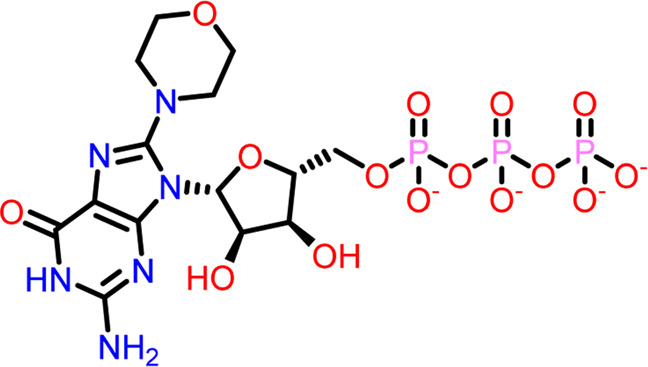	−9.276	Hydrogen Bond- Gly21, Arg29, Ala71, Gly72, Ala73, Met105, Gly108, Thr109, Gly110, Thr133, Arg143
		**((2R,3S,4R,5R)-5-(2-amino-8-morpholino-6-oxo-1,6-dihydro-9H-purin-9-yl)-3,4-dihydroxytetrahydrofuran-2-yl)methyl triphosphate**		
5	Compound E	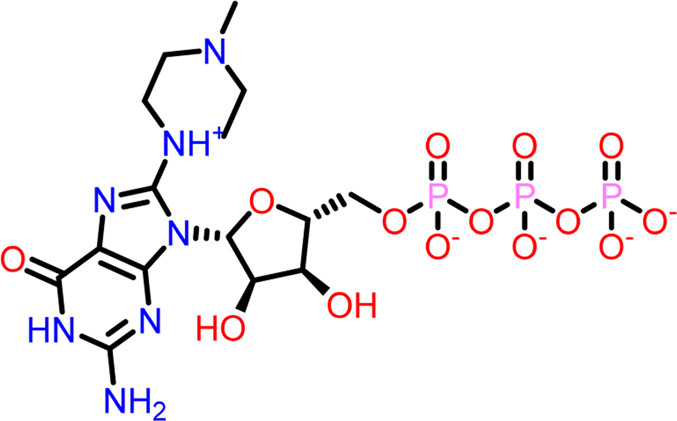	−9.244	Hydrogen Bond- Gly21, Gly22, Ala71, Ala73, Met105, Gly108, Thr109, Arg143
		**((2R,3S,4R,5R)-5-(2-amino-8-(4-methylpiperazin-1-ium-1-yl)-6-oxo-1,6-dihydro-9H-purin-9-yl)-3,4-dihydroxytetrahydrofuran-2-yl)methyl triphosphate**		
6	Compound F	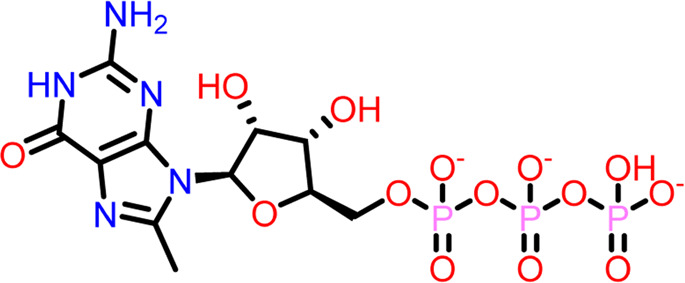	−8.902	Hydrogen Bond- Gly22, Asn25, Arg29, Ala71, Ala73, Gly108, Thr109, Thr133, Glu139, Arg143, Asn166
		**((2R,3S,4R,5R)-5-(2-amino-8-methyl-6-oxo-1,6-dihydro-9H-purin-9-yl)-3,4-dihydroxytetrahydrofuran-2-yl)methyl hydrogen triphosphate**		
7	Compound G	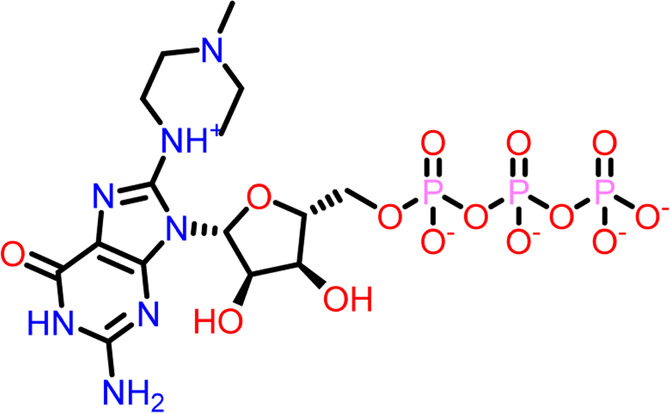	−8.900	Hydrogen Bond- Gly21, Gly22, Ala71, Ala73, Met105, Gly108, Thr109, Arg143
		**((2R,3S,4R,5R)-5-(2-amino-8-(4-methylpiperazin-1-ium-1-yl)-6-oxo-1,6-dihydro-9H-purin-9-yl)-3,4-dihydroxytetrahydrofuran-2-yl)methyl triphosphate**		
8	Compound H	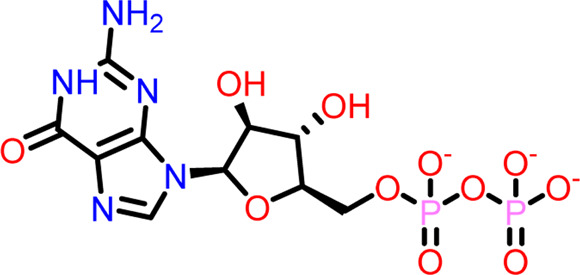	−8.886	Hydrogen Bond- Gly21, Gly22, Asn25, Arg29, Gly108, Thr109, Gly110, Thr133, Glu139, Arg143, Asn166
		**((2R,3S,4S,5R)-5-(2-amino-6-oxo-1,6-dihydro-9H-purin-9-yl)-3,4-dihydroxytetrahydrofuran-2-yl)methyl diphosphate**		
				Pi-Pi Stacking- Phe183
9	Compound I	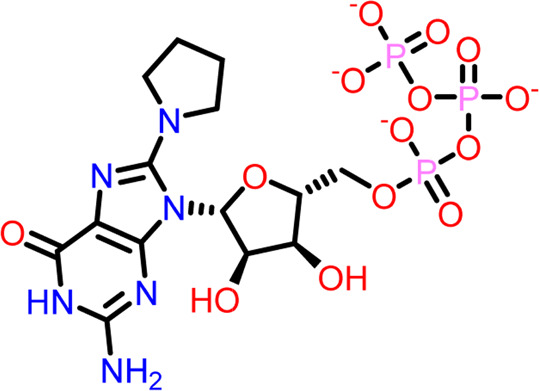	−8.837	Hydrogen Bond- Gly22, Asn25, Arg29, Gly108, Thr109, Gly110, Glu139, Arg143, Asn166
		**((2R,3S,4R,5R)-5-(2-amino-6-oxo-8-(pyrrolidin-1-yl)-1,6-dihydro-9H-purin-9-yl)-3,4-dihydroxytetrahydrofuran-2-yl)methyl triphosphate**		Salt Bridge- Arg143
				Pi-Pi Stacking- Phe183
10	Compound J	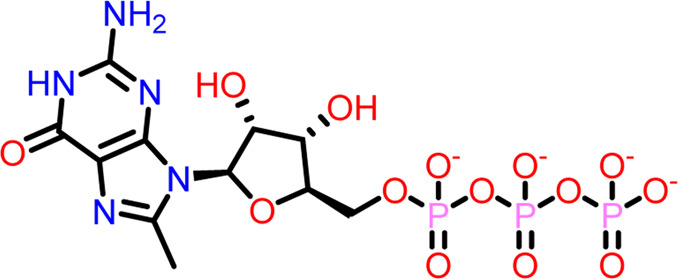	−8.789	Hydrogen Bond- Gly22, Asn25, Arg29, Ala71, Ala73, Gly108, Thr109, Glu139, Arg143, Asn166
		**((2R,3S,4R,5R)-5-(2-amino-8-methyl-6-oxo-1,6-dihydro-9H-purin-9-yl)-3,4-dihydroxytetrahydrofuran-2-yl)methyl triphosphate**		Pi-Pi Stacking- Phe183
11	Control drug	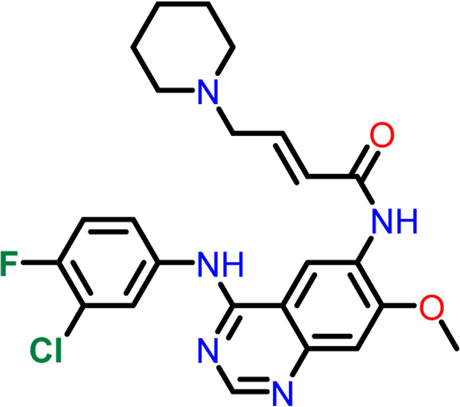	−4.450	Hydrogen Bond- Gly22, Thr109, Arg143
		**Dacomitinib**		Salt Bridge- Glu139

**FIGURE 5:**
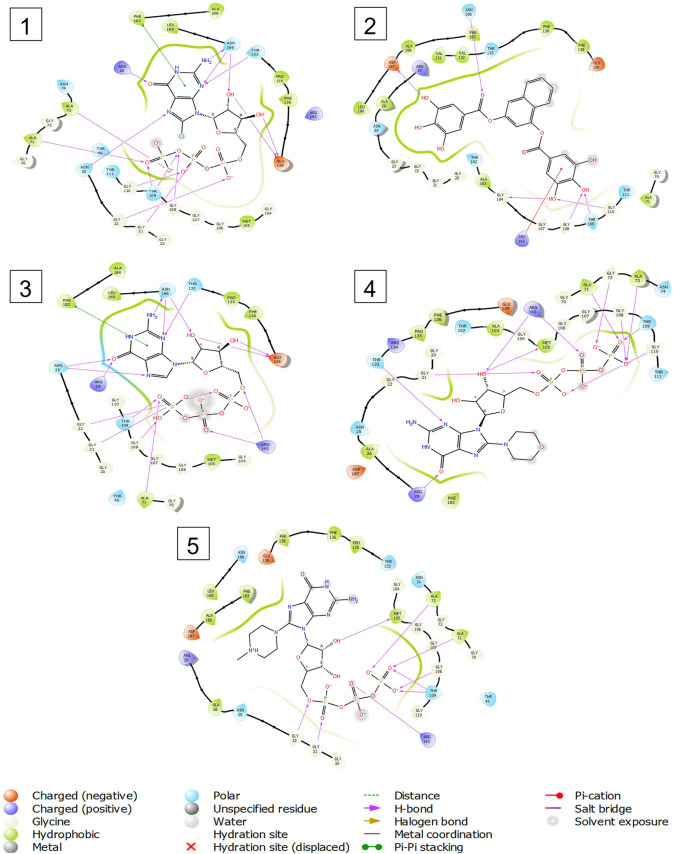
2-D interaction diagrams of top five docking score ligand-protein complexes at nucleotide binding site. The purple, green and red arrows represents hydrogen bonding, Pi-Pi stacking and Pi-cation interactions between ligands and FtsZ protein, (1) Compound A [-9.549 kcal/mol], (2) Compound B [-9.539 kcal/mol], (3) Compound C [-9.530 kcal/mol], (4) Compound D [-9.276 kcal/mol], (5) Compound E [-9.244 kcal/mol].

**FIGURE 6:**
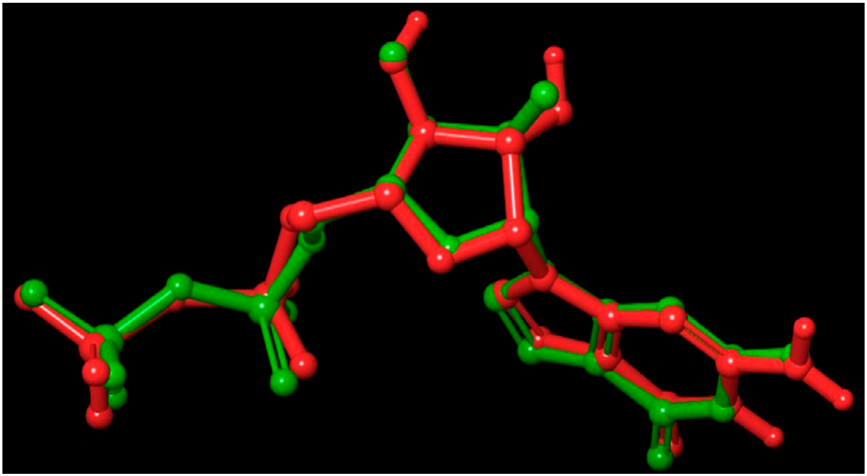
Validation of the molecular docking procedure used by using the before docking (green) and after docking (red) poses of co-crystallized ligand (GDP). Both the poses of GDP overlaps almost exactly with an RMSD of 0.4302, showing the validity of our docking method and the accuracy of all docking scores.

Out of 138 screened molecules, Compound A (2R,3S,4R,5R)-5-(2-amino-8-chloro-6-oxo-1,6-dihydro-9H-purin-9-yl)-3,4-dihydroxytetrahydrofuran-2-yl)methyl triphosphate) i. e, chloro derivative of GTP had the highest docking score of −9.549 kcal/mol compared to Compound I ((2R,3S,4S,5R)-5-(2-amino-6-oxo-1,6-dihydro-9H-purin-9-yl)-3,4-dihydroxytetrahydrofuran-2-yl)methyl diphosphate i.e., GDP molecule, a co-crystallized ligand which was found in the template protein, with a docking score of −8.886 kcal/mol. Following analysis of the 2D interaction diagram of the top four ligand-protein complex, it was found that while the ring structure of the GTP derivative produced a Pi-Pi stacking interaction with the aromatic amino acid Phenylalanine (Phe) 183 of active site, phosphate groups interacted with the majority of amino acids by forging hydrogen bonds Figure 5. (Figure 5A). Compound B produced hydrogen bonds and pi-cation interactions with the amino acids Gly104, Gly108, Thr109, Gly110, Asn166, Asp187, and Arg143, respectively (Figure 5B). It was observed that the compound C have shown similar interactions as that of compound A. (Figure 5C). The Compound D has interacted with Gly21, Arg29, Ala71, Gly72, Ala73, Met105, Gly108, Thr109, Gly110, Thr133, and Arg143 residues *via* hydrogen bonding (Figure 5D). whereas the compound E formed hydrogen bonding interaction with Gly21, Gly22, Ala71, Ala73, Met105, Gly108, Thr109, and Arg143 residues (Figure 5E). in the nucleotide binding site. The hydrogen bond interactions with Gly108 was found common in both GTP and non GTP derivatives which can be assumed as a crucial interaction for inhibiting FtsZ activity. The control drug i.e., Dacomitinib has interacted with Gly22, Thr109, Arg143 *via* hydrogen bonding and Salt Bridge with Glu139 of FtsZ’s nucleotide binding site. The docking results were validated by redocking the co-crystallized ligand (GDP) in the template protein (PDB—4M8I) to its nucleotide binding site. The co-crystal ligands’ original and docked confirmations were compared, and the computed root means square deviation (RMSD) between them was less than 2 Å i.e. 0.4302 Å (Figure 6).

### Molecular docking at inter-domain binding site

Small molecules bind to the region between the N and C-terminal guanosine triphosphate (GTP) binding subdomains i.e., the inter-domain binding site of FtsZ, reducing its capacity to function allosterically, which eventually prevents bacterial division. However, the lack of adequate chemical tools to develop a binding screen against this region has hindered the search for FtsZ antibacterial inhibitors. (Huecas et al., 2021).

At the FtsZ protein’s inter-domain binding site, ligand docking was conducted using the ligand molecules taken from the literature. The top 10 outputs’ binding affinities range was −7.243 to −4.290 kcal/mol (Table 2). According to the findings of this study, the ligands interact with the active residues Asp199, Glu301, Asn263, Thr265, Glu192, Thr309, and Gly196 through interactions that entail both salt bridges and hydrogen bonds.

**TABLE 2:** Molecular docking results of top 10 hits at Inter-domain binding site.

S.No	Ligands	Structure	Docking score (kcal/mol)	Interaction
1	Compound 1	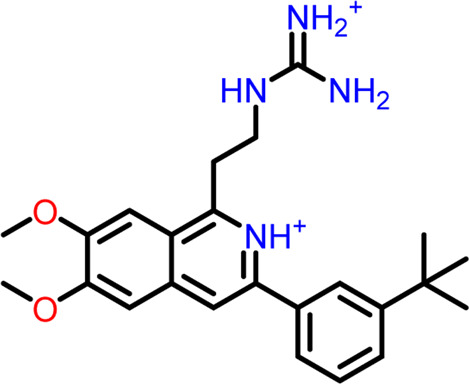 **1-(2-((amino**(**iminio)methyl**)**amino)ethyl**)**-3-(3-(tert-butyl)phenyl**)**-6,7-dimethoxyisoquinolin-2-ium**	-7.243	Salt Bridge- Asp199
				Hydrogen Bond- Glu301
2	Compound 2	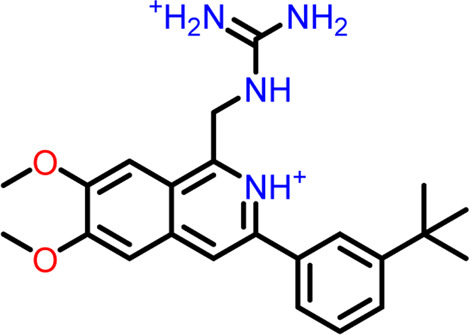 **1-(((amino**(**iminio)methyl**)**amino)methyl**)**-3-(3-(tert-butyl)phenyl**)**-6,7-dimethoxyisoquinolin-2-ium**	-6.518	Salt Bridge- Asp199
				Hydrogen Bond- Glu301
3	Compound 3	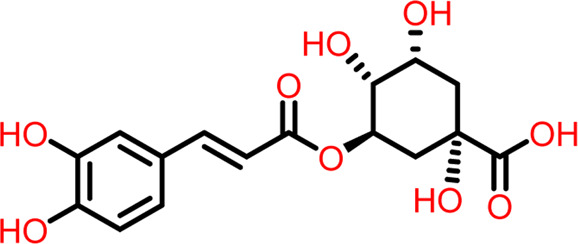 **Chlorogenic Acid**	-6.355	Salt Bridge- Arg191
				Hydrogen Bond- Asn263, Thr265
4	Compound 4	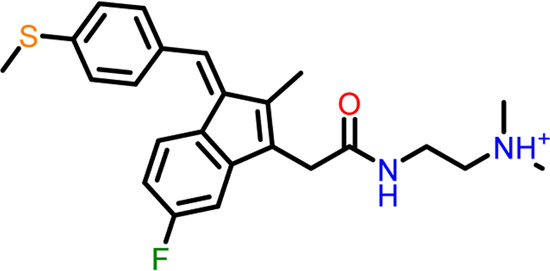 **(Z)-2-(2-(5-fluoro-2-methyl-1-(4-(methylthio)benzylidene**)**-1H-inden-3-yl)acetamido**)**-N,N-dimethylethan-1-aminium**	-5.376	Hydrogen Bond - Asp199
5	Compound 5	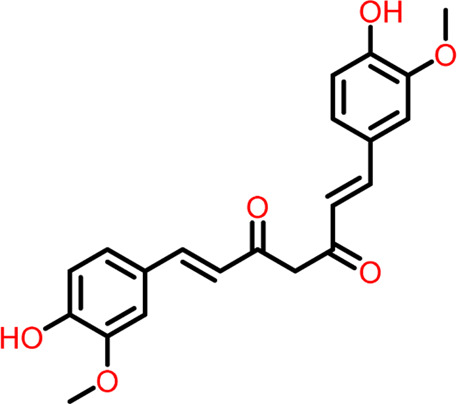 **Curcumin**	-5.273	Hydrogen Bond- Arg191, Glu192, Asn263
6	Compound 6	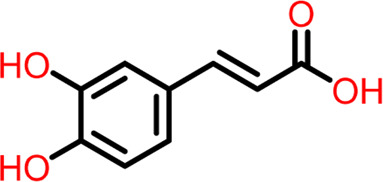 **Caffeic Acid**	-5.097	Hydrogen Bond- Thr309
7	Compound 7	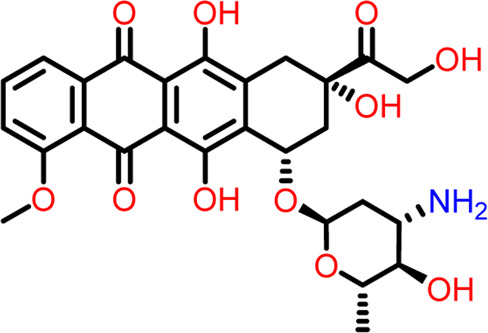 **Epirubicin**	-5.067	Salt Bridge- Asp199, Thr309, Gly196
				Hydrogen Bond- Glu301
8	Compound 8	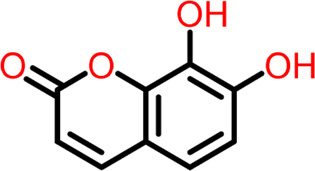 **Daphnetin**	-5.057	Hydrogen Bond- Thr309
9	Compound 9	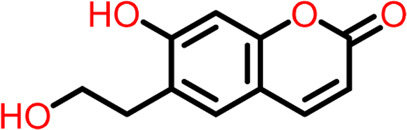 **Phellodenol A**	-4.408	Hydrogen Bond- Asn263, Thr265, Val307
10	Compound 10	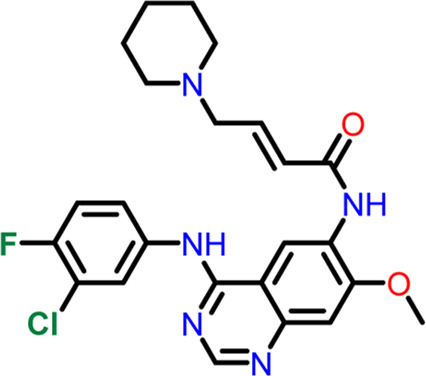 **(E)-2-(2-(1-(benzo**[**d]thiazol-2-ylmethylene)-5-fluoro-2-methyl-1H-inden-3-yl)acetamido**)**-N,N-diethylethan-1-aminium**	-4.290	Salt Bridge & Hydrogen Bond- Asp199
11	Control drug (PC190723)	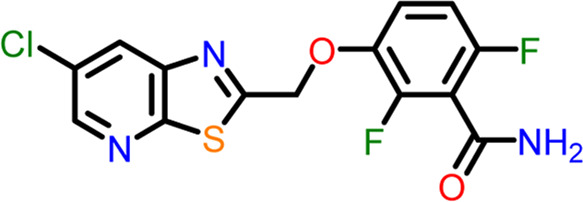 **3-((6-chlorothiazolo[5,4-b]pyridin-2-yl)methoxy**)**-2,6-difluorobenzamide**	-4.694	Halogen Bond –Asn25, Arg29
				Hydrogen Bond- Gly108, Thr109, Thr133, Asn166

**FIGURE 7:**
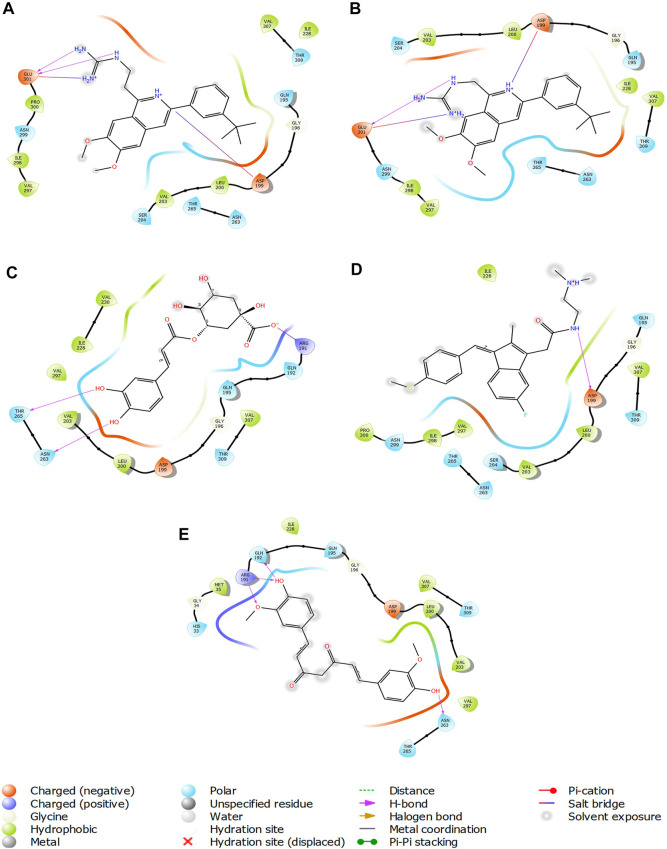
2-D interaction diagrams of top five ligand-protein complexes at inter-domain binding site, the interaction in purple colour represents hydrogen bonding, while blue-red line depicts salt bridge interaction between ligand and protein residues, **(A)** Compound 1 [-7.243 kcal/mol], **(B)** Compound 2 [-6.518 kcal/mol], **(C)** Compound 3 [-6.355 kcal/mol], **(D)** Compound 4 [-5.376 kcal/mol], **(E)** Compound 5 [-5.273 kcal/mol].

The two-dimensional interaction diagram of the ligand-protein complex at this binding site is shown in (Figure 7). While Glu301 formed hydrogen connections with Compounds 1 and 2, Asp199 altered the salt bridge (Figures 7A,B). Compound 3 (chlorogenic acid) formed hydrogen bonds with Asn263, Thr265, and Asp199 (Figure 7C). Compound 4 has shown only hydrogen bond interaction with Asp199 (Figure 7D). Compound 5 has interacted with Arg191, Glu192, Asn263 residues *via* hydrogen bonding (Figure 7E). The interaction with Asp199 *via* hydrogen bonding or through salt bridge was found to be common among all the hits. This indicates that the interaction with Asp199 may be essential for the compounds to exhibit FtsZ inhibitory activity.

### Prime MM/GBSA analysis of docked complexes at nucleotide binding site

The binding free energies of the top five docked complexes were calculated using the MM/GBSA approach. The results revealed that the binding free energy of FtsZ’s nucleotide-binding site with compounds A, B, C, D and E were found to be −41.92, −58.09, −65.68, −34.80, and −26.90 kcal/mol, respectively (Table 3). According to the total binding free energies, the hit molecules may form a potent interaction within the binding region of the selected target, thereby inhibiting enzyme activity.

**TABLE 3:** Binding free energies of top five compounds in complex with nucleotide binding site.

Compound ID	MM/GBSA dG bind (kcal/mol)	MM/GBSA dG bind coulomb (kcal/mol)	MM/GBSA dG bind covalent (kcal/mol)	MMGBSA dG bind Hbond (kcal/mol)	MMGBSA dG bind Vdw (kcal/mol)	MMGBSA dG bind packing (kcal/mol)	MMGBSA dG bind solv GB (kcal/mol)
Compound A	−41.92	44.80	11.48	−14.19	−43.39	−3.24	−31.89
Compound B	−58.09	−45.60	6.90	−5.07	−45.17	−0.74	46.59
Compound C	−65.68	20.61	3.01	−13.72	−41.78	−2.80	−25.83
Compound D	−34.80	53.73	10.56	−12.60	−53.21	−3.21	−25.56
Compound E	−26.90	64.86	9.37	−9.90	−57.21	−1.54	−22.30
Dacomitinib	−28.01	−11.85	8.83	−4.04	−43.86	−0.98	35.49

### Prime MM/GBSA analysis of docked complexes at inter-domain binding site

The binding free energies of the top five docked complexes were calculated using the MM/GBSA approach. The results revealed that the binding free energy of FtsZ’s nucleotide-binding site with compounds 1, 2, 3, 4, and 5 was found to be −49.14, −48.19, −19.67, −28.92, and −40.51 kcal/mol, respectively (Table 4). These hits may generate a strong interaction within the binding region of the chosen target, according to the total binding free energies, which would impede enzyme activity.

**TABLE 4:** Binding free energies of top 5 compounds in complex with inter-domain binding site.

Compound ID	MM/GBSA dG bind (kcal/mol)	MM/GBSA dG bind coulomb (kcal/mol)	MM/GBSA dG bind covalent (kcal/mol)	MM/GBSA dG bind Hbond (kcal/mol)	MM/GBSA dG bind Vdw (kcal/mol)	MM/GBSA dG bind packing (kcal/mol)	MM/GBSA dG bind solv GB (kcal/mol)
Compound 1	−49.14	−339.49	4.71	−1.93	−34.07	−3.74	340.74
Compound 2	−48.19	−335.37	2.56	−1.47	−33.56	−3.71	338.33
Compound 3	−19.67	124.94	7.49	−2.22	−30.16	−0.03	-108.22
Compound 4	−28.92	−84.45	1.22	−0.83	−31.90	−1.57	102.28
Compound 5	−40.51	−22.87	3.83	−2.60	−35.15	−0.31	32.22
PC190723	−36.51	−29.75	2.16	−2.86	−33.58	−1.78	38.15

### ADME/T prediction of top hits

ADME parameters were predicted for the top 5 hits of the nucleotide-binding site (Table 5). It was noticed that although most of the successful compounds were predominantly nucleotide analogues, which have shown excellent results, their expected oral absorption percentage in humans was low. The top 10 hit molecules were anticipated to be non-toxic compounds, as determined by Protox II (Table 6).

**TABLE 5:** Physicochemical and pharmacokinetic parameters of top five hits obtained after docking at nucleotide binding site.

SI. No	Compound	Mol wt	Donor HB	Acceptor HB	QPlogS	QPlogP o/w	Percent oral human absorption	QPPCaCO	QPlogBB
1	Compound A	557.628	5.000	21.600	-0.931	−3.026	0.000	0.001	−5.812
2	Compound B	464.384	6.000	9.500	−4.477	0.694	26.600	3.003	−4.142
3	Compound C	523.183	5.000	21.600	−0.590	−3.234	0.000	0.001	−5.634
4	Compound D	608.288	14.934	23.800	−0.704	−3.068	0.000	0.001	−5.613
5	Compound E	621.330	5.000	24.100	−0.604	−4.864	0.000	0.001	−4.970

**TABLE 6:** Toxicity results of top hit molecules screened at nucleotide binding site of Ftsz.

SI.No	Compound ID	Predicted toxicity class	Predicted LD50 (mg/kg)	Organ toxicity	Toxicity end points
1	Compound A	Class V	3790 mg/kg	-	-
2	Compound B	Class V	2260 mg/kg	-	-
3	Compound C	Class V	3790 mg/kg	-	-
4	Compound D	Class V	3790 mg/kg	-	-
5	Compound E	Class V	3790 mg/kg	-	-

Following docking to the inter-domain binding site of FtsZ, the top 5 hits were predicted for their ADME properties (Table 7). The majority of the compounds have shown encouraging findings, and it was found that the projected values fall within the acceptable range. Although none of the compounds shows organ toxicity, Protox II predicted toxicity endpoints like immunotoxicity. (Table 8).

**TABLE 7:** Physicochemical and pharmacokinetic parameters of top hits obtained after docking at inter-domain binding site.

SI. No	Compound	Mol wt	Donor HB	Acceptor HB	QPlogS	QPlogPo/w	Percent oral human absorption	QPPCaCO	QPlogBB
1	Compound 1	406.527	4.000	4.500	−6.040	4.608	100.000	804.316	−1.107
2	Compound 2	392.500	4.000	4.500	−6.010	4.497	100.000	793.324	−1.043
3	Compound 3	354.313	6.000	9.650	−2.380	−0.231	19.069	2.288	−3.141
4	Compound 4	410.548	1.000	5.000	−4.890	4.709	100.000	418.103	0.108
5	Compound 5	368.385	2.000	7.000	−4.240	2.831	85.688	226.891	−1.989

**TABLE 8:** Toxicity results of top hit molecules of inter-domain binding site screening.

SI.No	Compound ID	Predicted toxicity class	Predicted LD50 (mg/kg)	Organ toxicity	Toxicity end points
1	Compound 1	Class III	200 mg/kg	**-**	Immunotoxicity
2	Compound 2	Class III	200 mg/kg	**-**	Immunotoxicity
3	Compound 3	Class V	5000 mg/kg	-	Immunotoxicity
4	Compound 4	Class III	200 mg/kg		Immunotoxicity
5	Compound 5	Class IV	2000 mg/kg	-	Immunotoxicity

### Molecular dynamics simulation

To investigate the real-time dynamics and conformational stability of a protein upon binding to a specific ligand, 100 ns of MD simulations were performed on the five best-docked compounds, (A, B, C, D, and E) at nucleotide binding site and (compound 1, 2, 3, 4, and 5) at inter-domain binding site. Our analysis of simulation interaction diagrams (SIDs) for the 100-ns SPC water model-based simulations provided a better understanding of Protein RMSD, Ligand RMSD, Protein RMSF, Ligand RMSF, Protein-Ligand contacts, and ligand characteristics were analyzed, and a simulation interaction diagram was constructed to assess the stabilities of the protein-ligand complexes.

### RMSD of top five hits at nucleotide binding site

**FIGURE 8:**
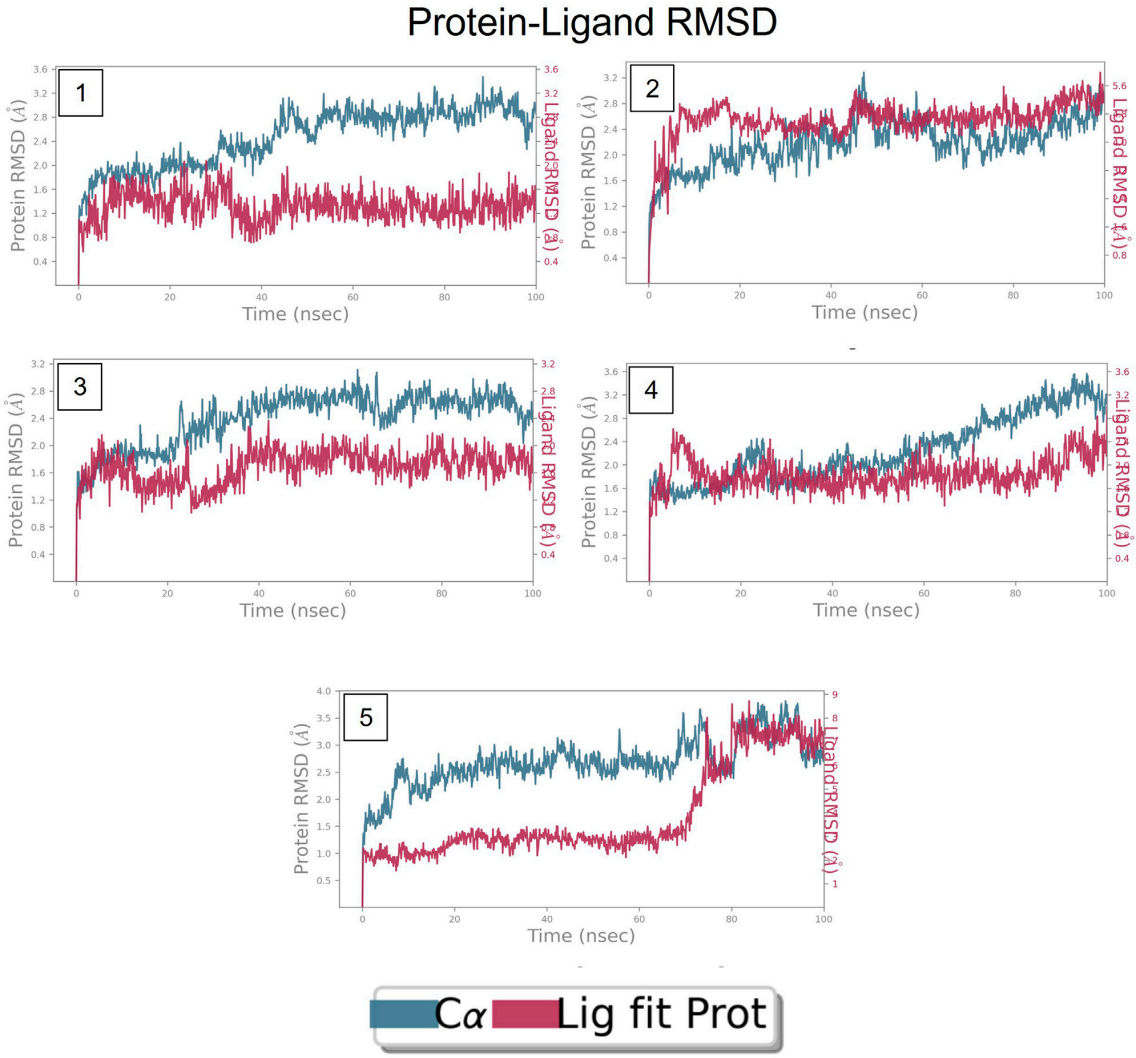
RMSD plot of FtsZ protein in complex with Compounds at nucleotide binding site during period of MD simulation, Blue line in graph shows protein RMSD in form of C alpha chain of protein and pink lines represents Lig fit Prot RMSD, protein RMSD values are on left *y*-axis while lig fit prot RMSD are on right *Y*-axis, the X-axis shows time period of MD simulation in nanoseconds. (1) RMSD of Compound A, (2) RMSD of Compound B, (3) RMSD of Compound C, (4) RMSD of Compound D, and (5) RMSD of Compound E.

The RMSD is generally used to calculate protein backbone (Cα, C, and N) deviation during the 100 ns simulation period. The FtsZ bound to compound A (Chloro derivative of GTP) showed a protein RMSD of 1.6–3.2 Å and ligand RMSD of 1.1–2.0 Å. Convergence was observed between the two plots around the initial 0–35 ns, which indicates the FtsZ-Chloro derivative of GTP complex’s stability. Additionally, for ligand, slight fluctuation in RMSD, was observed between 35–45 ns of trajectory; however, it remained consistent after that with RMSD of 0.6–1.8 Å Figure 8 (Figure 8A). In case of Compound B (naphthalene-1,3-diyl bis(3,4,5-trihydroxybenzoate)-FtsZ complex, the protein and ligand RMSD plots slightly fluctuated during the initial 0–20 ns and remained converged throughout the simulative period with RMSD of 1.8–5.6 Å which can be inferred as a stable complex (Figure 8B). The compound C-FtsZ complex MD trajectory of 100 ns indicates that the complex tends to be stable during simulation with a RMSD of less than 3 Å. This indicates that the FtsZ- Guanosine triphosphate (GTP) complex did not lead to much conformational changes in the dynamics environment (Figure 8C). The ligand RMSD of compound D bound to FtsZ was found to be in the range of 2.0–2.4 Å, and the protein’s RMSD lay in the range of 1.8–3.2 Å. Initially, the slight fluctuation was observed in the trajectory at 0–15 ns time frame followed by the convergence of both the RMSDs till 60 ns, a slight change in order was observed at 70–100 ns, which falls in the acceptable range (Figure 8D). This ultimately indicates the FtsZ-morpholine derivative of GTP complex’s stability. The FtsZ protein’s RMSD was 1.8–7.8 Å, whereas compound E’s RMSD was 1.1–8.0 Å (methylpiperazine derivative of GTP). Even though the RMSD plots of the ligand and the protein converged for a certain period, after 70 ns, the complex was entirely out of the binding pocket when the simulation trajectory was observed. As the ligand was found to show significant variations in their RMSD, compound E is considered an unstable molecule (Figure 8E).

### RMSD of top five hits at inter-domain binding site

**FIGURE 9:**
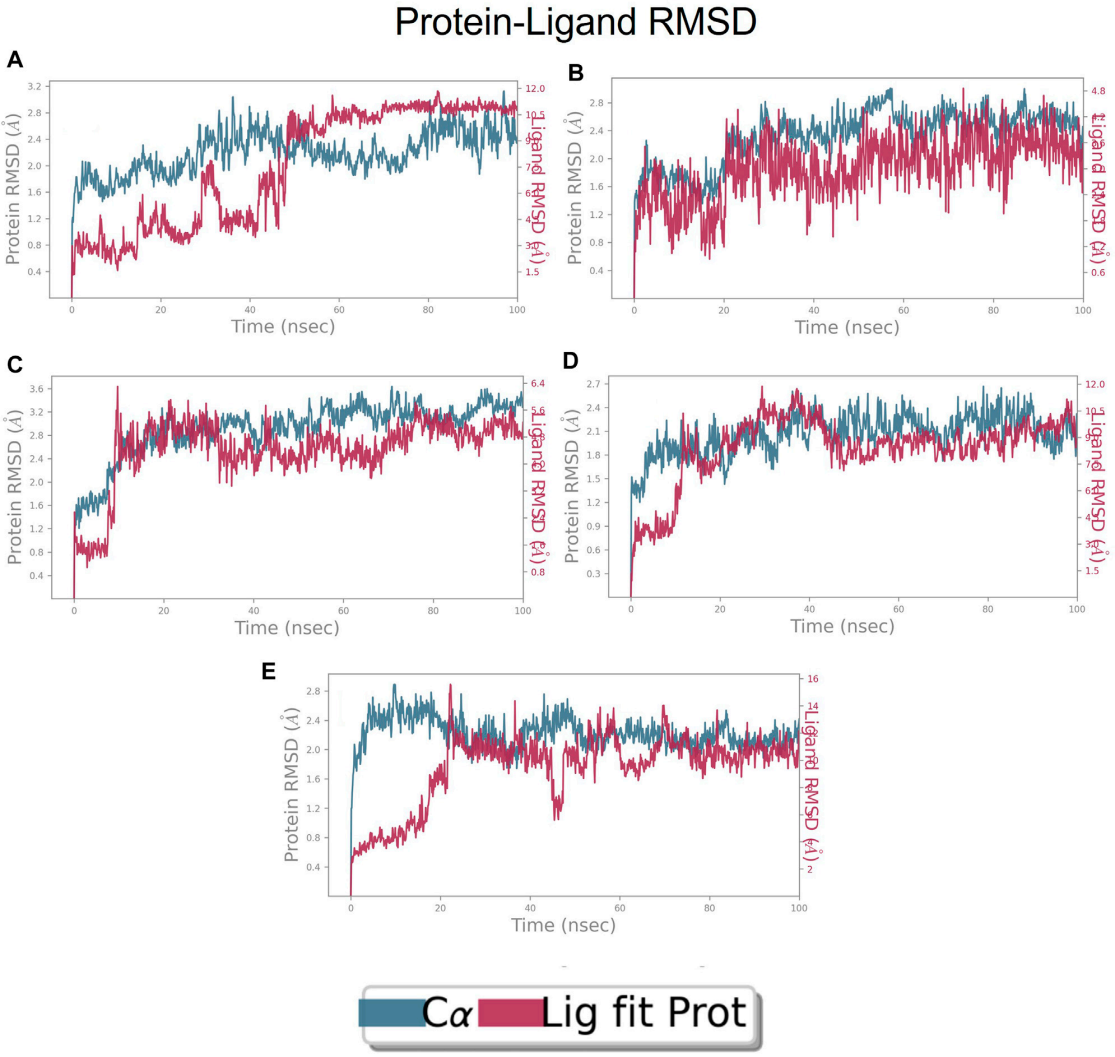
RMSD plot of FtsZ protein in complex with Compounds at inter-domain binding site during period of MD simulation, Blue line in graph shows protein RMSD in form of C alpha chain of protein and pink lines represents Lig fit Prot RMSD, protein RMSD values are on left *y*-axis while lig fit prot RMSD are on right *Y*-axis, the X-axis shows time frame of MD simulation trajectory in nanoseconds. **(A)** RMSD of Compound 1 **(B)** RMSD of Compound 2, **(C)** RMSD of Compound 3 **(D)** RMSD of Compound 4, and **(E)** RMSD of Compound 5.

The RMSD of the protein in the presence of compound 1 [1-(2-((amino(iminio)methyl)amino)ethyl)-3-(3-(tert-butyl)phenyl)-6,7-dimethoxyisoquinolin-2-ium] was found to be 1.8–10.5 Å whereas the ligand RMSD was in the range of 0.8–12 Å (Figure 9 (Figure 9A). Variation in both protein and ligand RMSDs was recorded until the end of the simulation, indicating that the ligand diffused away from the binding and complex is unstable. The compound 2 RMSD plot converged well with the FtsZ protein backbone for the 100 ns simulation period with a RMSD of 2.3–4.2 Å indicating ligand’s tight binding to the protein’s binding pocket and hence a stable complex (Figure 9B). In the case of compound 3 [chlorogenic acid], the RMSDs of the FtsZ protein and ligand were found fluctuating for the initial 0–10ns, but were found to show consistent stability with a ligand RMSD of less than 3.6–4.8 Å and protein RMSD was in the range of 2.9–6.2 Å (Figure 9C). The protein RMSD was found to be in the range of <3 Å in the case of both compounds 4 [(Z)-2-(2-(5-fluoro-2-methyl-1-(4-(methylthio)benzylidene)-1H-inden-3-yl)acetamido)-N,N-dimethylethan-1-aminium] and compound 5 [Curcumin], but the ligand RMSDs was found to be 0.9–10.5 Å and 0.5–10 Å respectively. Due to the large deviation among the protein and ligand RMSDs, the compound 4-FtsZ complex was found to be unstable throughout the 100ns simulation (Figure 9D) and when the simulation trajectory was observed, the ligand protruded away after 20 ns from the binding pocket indicating poor stability with FtsZ. The compound 5-FtsZ complex was unstable during the 100 ns simulation due to a substantial discrepancy between the protein and ligand RMSDs (Figure 9E).

Based on the RMSDs of complexes, the stable complexes were considered for further analysis of simulation parameters like RMSF of protein and ligand, protein-ligand contacts and changes in the ligand properties during simulation.

### Protein and ligand RMSF at nucleotide binding site

**FIGURE 10:**
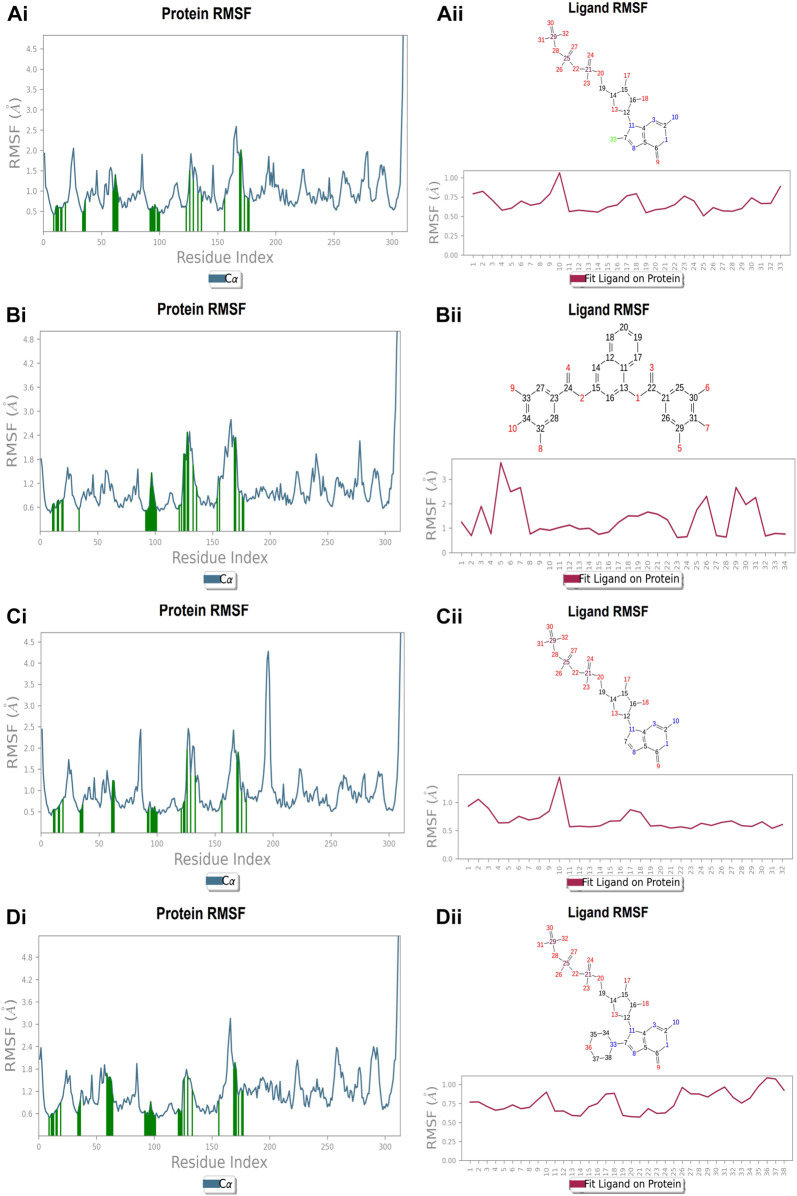
RMSF plot of Ftsz Protein where the active site residues are on X-axis, root mean square fluctuation (RMSF) values on Y-axis, the green bar indicate point of contact of respective amino acid residues at nucleotide binding site with the following ligands (Ai) Compound A, (Bi) Compound B, (Ci) Compound C, and (Di) Compound D; Ligand RMSF plots of the compounds (Aii) Compound A, (Bii) Compound B, (Cii) Compound C, and (Dii) Compound D at nucleotide binding site. The pink line in the plot shows RMSF of ligand, the X-axis indicates residues of ligand and Y-axis shows value of RMSF.

**FIGURE 11:**
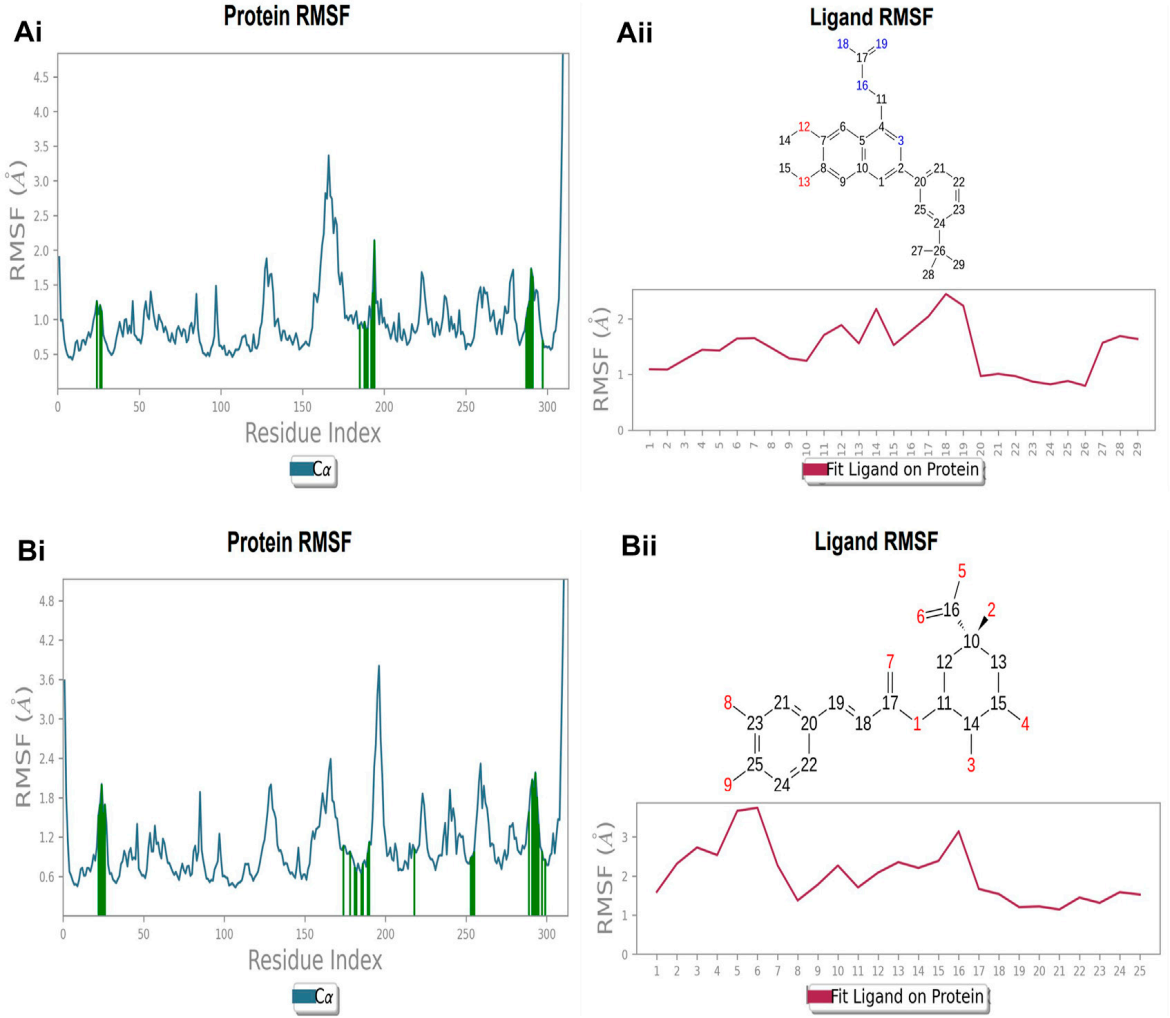
RMSF plot of Ftsz Protein where the active site residues are on X-axis, root mean square fluctuation (RMSF) values on Y-axis, the green bar indicate point of contact of respective amino acid residues at inter-domain binding site with the following ligands (Ai) Compound 2, and (Bi) Compound 3; Ligand RMSF plots of the compounds (Aii) Compound 2, (Bii) Compound 3 at inter-domain binding site. The pink line in the plot shows RMSF of ligand, the X-axis indicates residues of ligand and Y-axis shows value of RMSF.

**FIGURE 12:**
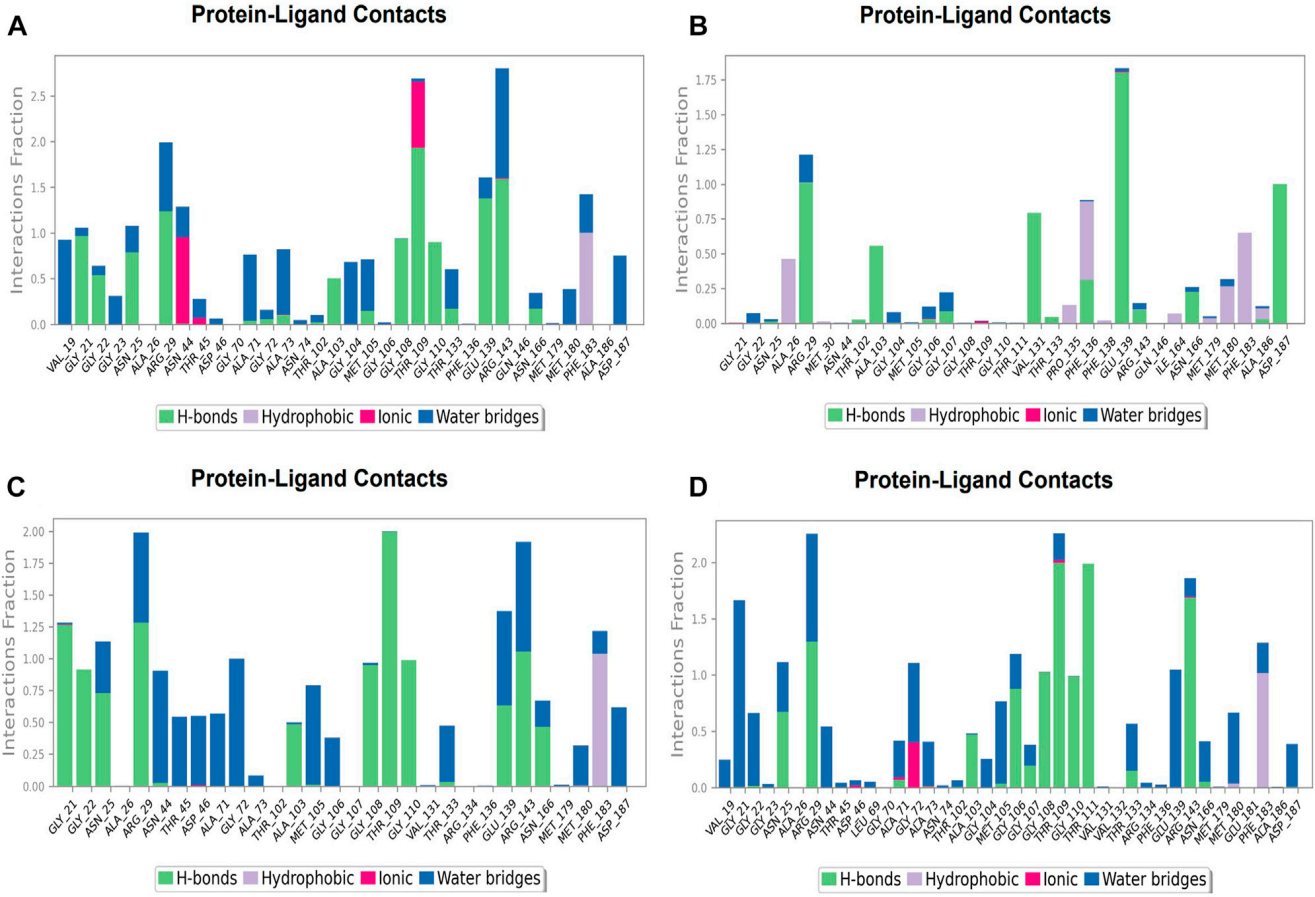
Protein-ligand Contacts histogram shows ligand interaction with amino acids at nucleotide binding site, purple for hydrophobic interaction, green for hydrogen bond, pink for ionic interaction whereas blue for water bridge, X-axis indicate amino acid residues while Y-axis shows Interaction fraction **(A)** Compound A, **(B)** Compound B, **(C)** Compound C, and **(D)** Compound D.

**FIGURE 13:**
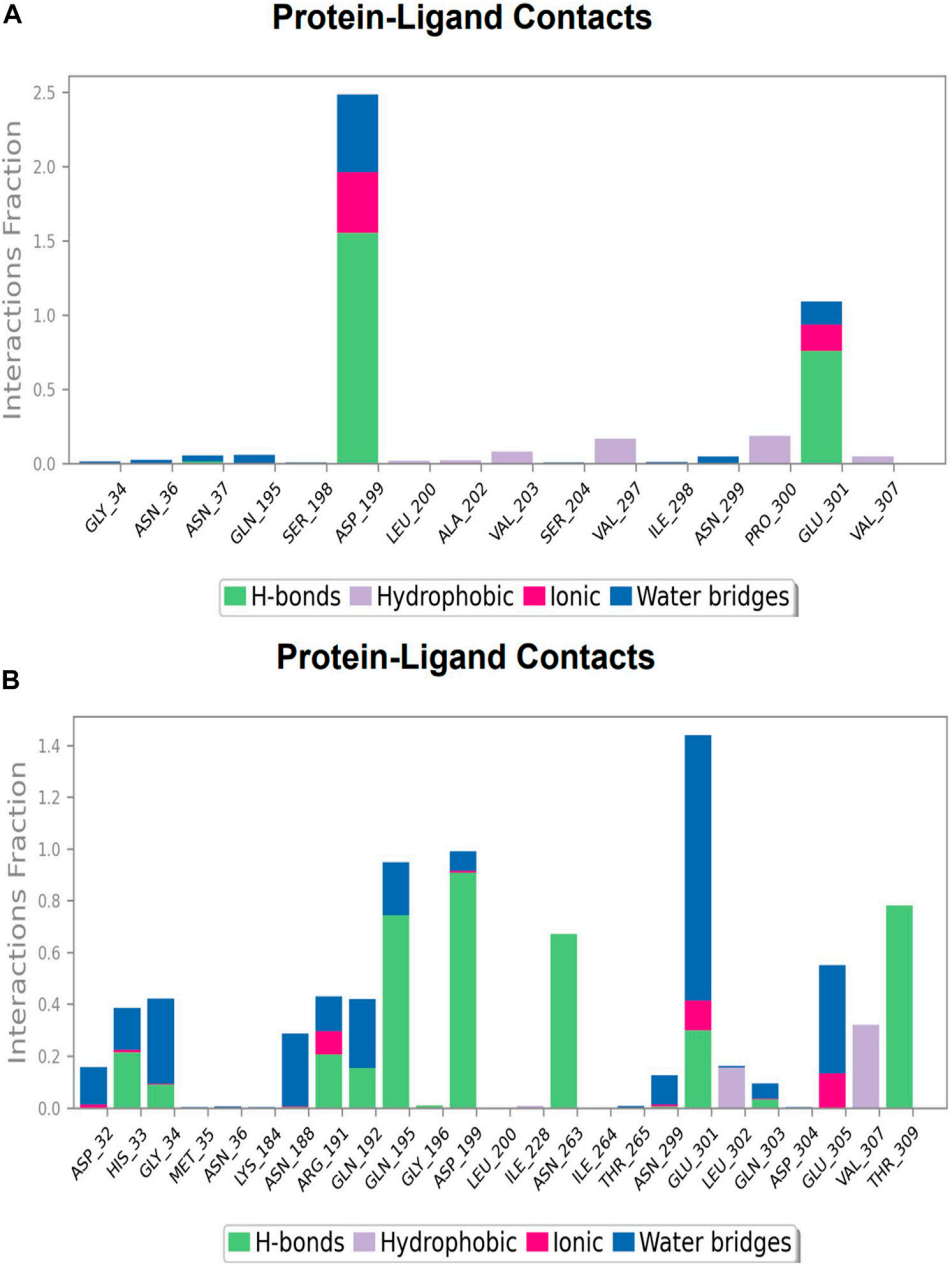
Protein-ligand Contacts histogram shows ligand interaction with amino acids at inter-domain binding site, purple for hydrophobic interaction, green for hydrogen bond, pink for ionic interaction whereas blue for water bridge, X-axis indicate amino acid residues while Y-axis shows Interaction fraction **(A)** Compound 2, and **(B)** Compound 3.

Each protein residue’s flexibility and mobility are represented by the RMSF value. Greater RMSF values suggest more flexibility throughout the MD simulation, while a lower RMSF value affects the system’s stability. The protein’s RMSF in the presence of compound A was found to be 0.5–2.0 Å Figure 10 (Figure 10A). Similarly, compound A did not show any major fluctuations in their RMSF, while element number 10 of the ligand (involved in making hydrogen bond interactions with the active site) exhibited small fluctuation, which is under the acceptable range, retains the stability of Compound A-FtsZ complex (Figure 10B). In the presence of compound B, the backbone residues demonstrated a substantially lower than permitted amount of fluctuation and was determined to be 0.8–2.4 Å Figure 11 (Figure 11A). The fluctuation was only observed at ligand’s element number 5, which is not engaged in making any contact with the active site (Figure 11B). According to the protein RMSF assessment results, the residues did not exhibit any observable flexibility in the presence of compound C, which ranged from 0.5 to 1.8 Å Figure 12 (Figure 12A). The ligand-protein complex may not result in any structural variation as the ligand’s RMSF was within the acceptable range (Figure 12B). In the presence of compound D, the protein’s RMSF was found to be 0.6 to 1.8 Å was the observed RMSF of protein which falls under the acceptable range and indicates the protein’s stability Figure 13 (Figure 13A). Variations in the ligand’s elements 36 and 37, which do not interact with the active site, may not lead to structural variability in the ligand-protein complex (Figure 13B).

### Protein and ligand RMSF at inter-domain binding site

The protein’s RMSF showed fewer fluctuations i.e., 1.2 and 2.4 Å in the presence of compound 2, indicating protein stability (Supplementary Figure S1A). There is slight fluctuation observed in the ligand’s RMSF among the elements 18 and 19, which are involved in forming hydrogen bonds with the active site in the ligand (Supplementary Figure S1B). This fluctuation may not affect the structural variability of the Compound 2-FtsZ complex as the observed RMSF was within the acceptable range. The RMSF of protein backbone residues in the inter-domain binding site vary from 1.9–2.0 Å, which is under the acceptable range (Supplementary Figure S2A), and the RMSF for Compound 3 was found to be 1.5–3.0 Å where the element numbers 5 and 6 of the ligand, which are not engaged in forming contacts with the active site, have no impact on the structural variation of the Compound 3-FtsZ complex (Supplementary Figure S2B).

### Protein-ligand contacts at nucleotide binding site

During the simulation phase, the atomic-level interaction information is critical for predicting the binding affinity of the protein and ligand. During the 100 ns simulation, intermolecular interactions between protein and ligand molecules, such as hydrogen bonds, ionic interactions, hydrophobic contact, and the salt bridge, were thoroughly investigated for binding analysis. The compound A was in contact with 34 amino acid residues in the nucleotide-binding domain, forming crucial interactions like hydrogen bonds, water bridges, ionic bonds and hydrophobic bonds (Figure 10C). 32 amino acid residues of the nucleotide-binding site came into contact with compound B during the simulation, creating critical interactions like hydrogen bonds, water bridges, ionic bonds, and hydrophobic bonds, most of which are hydrogen bond interactions (Figure 11C). Throughout the simulation period, 30 amino acid residues in the nucleotide-binding site came into contact with compound C, forming crucial interactions such as hydrogen bonds, water bridges, and hydrophobic bonds (Figure 12C). 40 amino acid residues in the nucleotide-binding domain interacted with chemical D, forming important interactions such as hydrogen bonds, water bridges, ionic bonds, and hydrophobic bonds (Figure 13C).

### Protein-ligand contacts at inter-domain binding site

During the course of a 100 ns simulation, 16 amino acid residues in the inter-domain binding site made contact with compound 2, forming significant interactions such as hydrogen bonds, water bridges, ionic bonds, and hydrophobic bonds, the majority of which are hydrogen bond interactions (Supplementary Figure S1C). Compound 3 generated significant interactions with 25 amino acid residues of FtsZ, including hydrogen bonds, water bridges, ionic bonds, and hydrophobic bonds, among others, the water bridges and hydrogen bonds making most of these interactions (Supplementary Figure S2C).

### Changes in the ligand properties during simulation

To assess the stability of the lead molecules in the nucleotide and inter-domain binding sites of the FtsZ, the five molecular characteristics of ligand (ligand RMSD, the radius of gyration [rGyr], Molecular surface area [MolSA], solvent accessible surface area [SASA], and polar surface area [PSA]) were also investigated.

#### Ligand RMSD

**FIGURE 14:**
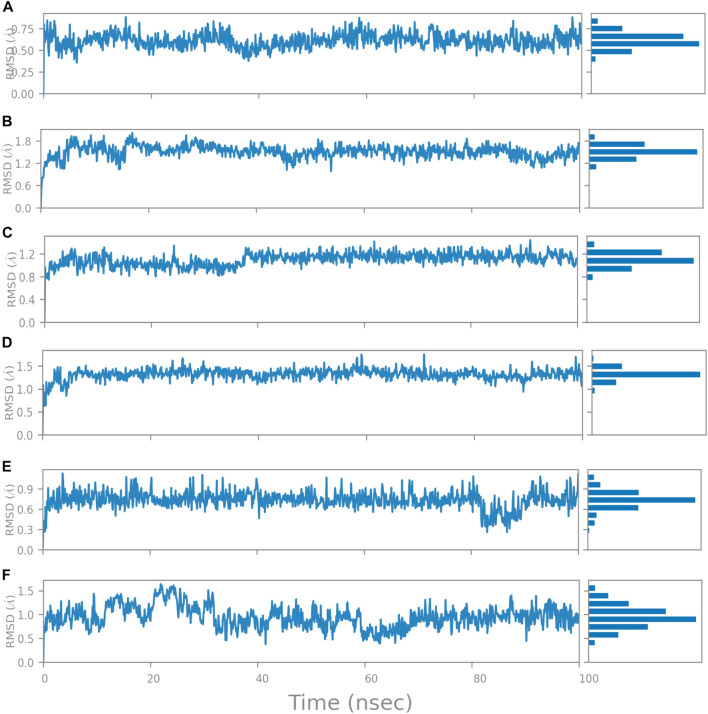
Change in Ligand RMSD’s during 100 ns simulation at nucleotide binding site **(A)** Compound A, **(B)** Compound B, **(C)** Compound C, and **(D)** Compound D; at inter-domain binding site **(E)** Compound 2, and **(F)** Compound 3.

“Ligand RMSD” refers to a ligand’s root mean square deviation from the reference conformation. Typically, the beginning frame is used as the reference and is treated as time t = 0. At the nucleotide-binding site, the RMSD of compound A was found below 0.75 Å (Figure 14A), whereas compound B was found between 1.2—1.8 Å (Figure 14B). Compounds C and D show RMSD between 0.8 (Figure 14C) and 1.2 Å, respectively (Figure 14D). In compounds 2 and 3 at the inter-domain binding site, the ligand RMSD was 0.3–0.9 Å (Figure 14E) and less than 1.5 Å (Figure 14F). All the molecules have shown the ligand RMSD value <2 Å indicating good ligand stabilities.

#### Radius of gyration

The radius of gyration is used to evaluate the ‘extendedness’ of a ligand and is comparable to its principal moment of inertia; the radius of gyration of compound A stayed constant during the 100 ns simulation and was found to be less than 5.0 Å indicating that the active site of a protein does not undergo any large conformational changes. The compound B’s rGyr was found to be 5.5 Å following 0–5 ns of stability without any significant conformational changes and retained the protein-ligand complex’s compactness. Compounds C and D, rGyr was found in the range 4.75–5.0 Å and 4.8–5.0 Å, which indicates the protein’s compactness during the simulation period. At the inter-domain binding site of FtsZ, the rGyr of Compound 2 was discovered to be steady and to lie between 4.64—4.72, Å Whereas compound 3, rGyr was found to be less than 4.6 Å after 45 ns of stabilization period because of protein-ligand complex compactness.

#### Molecular surface area (MolSA)

The compound A was found to be polar based on its MolSA, comparable to the van der Waals surface area, and was found to be 376–384 Å^2^. Because the MolSA of compound B was found to be 396–400 Å^2^, it can be assumed as a polar molecule. The compound C was found to be polar due to its MolSA ranging between 365 and 375 Å^2^_._ The compound D is polar, as evidenced by the MolSA value, which ranges from 420 to 440 Å^2^. At the inter-domain binding site, for compound 2, the MolSA value lies between 400 and 405 Å^2^, indicating its polarity. Compound 3 is polar, as evidenced by the MolSA, which was found to be 308–312 Å^2^.

#### Solvent-accessible surface area (SASA)

The SASA is the area of a molecule that the water molecules can access. The compound A has SASA in the range of 80–120 Å^2^. Higher SASA scores indicate that more of the molecule protrudes into the water, whereas lower scores indicate that the molecule is buried within the binding site. The SASA of compound B was determined to be 100–150 Å^2^, with an initial variation detected in the 0–5 ns time frame of MD simulation, indicating that the molecule is buried within the binding site. The compound C and D, SASA were between 80 and 120 Å^2^ and 120 to 180 Å^2^ respectively, indicating that more of the molecule is extending out into the water, which means good SASA.

#### Polar surface area (PSA)

The PSA is the solvent-accessible surface area solely contributed by oxygen and nitrogen atoms. Due to the presence of oxygen and nitrogen atoms in compound A, its PSA was observed between 540–550 Å^2^. The ligands with PSA >140 Å^2^ indicate good oral and intestinal absorption (Supplementary Figure S4A). Compound B may exhibit good oral and intestinal absorption with a PSA of 360–372 Å^2^ due to oxygen atoms in its structure (Supplementary Figure S4B). Compound C’s PSA of 540–555 Å^2^, signifying favourable oral and intestinal absorption, was established by the compound’s quantity of nitrogen and oxygen atoms (Supplementary Figure S4C **)**. Despite initial fluctuations at 0–5 ns, Compound D’s PSA of 520–540 Å^2^ was maintained until the 100 ns simulation, indicating that the amount of nitrogen and oxygen atoms in the compound determined its favourable oral and intestinal absorption (Supplementary Figure S4D). At the inter-domain binding site, compound 2’s PSA of 152–160 Å2 persisted through the 100 ns simulation, showing that the compound’s nitrogen and oxygen content led to the compound’s successful oral and intestinal absorption (Supplementary Figure S4E). The molecular polar surface area of compound 3 (Supplementary Figure S4F) was found to be 330–345 Å2 as it contains only oxygen atoms but not nitrogen atoms in its structure which generally corresponds to the accessibility towards the solvents present in the binding site.

The Compounds A, B, C, and D were potential lead molecules binding at the nucleotide-binding site, whereas compound 2 and 3 were considered potential inhibitors for the inter-domain binding sites. These lead compounds were further subjected to post MM/GBSA analysis to understand their binding strengths.

### Post MM/GBSA analysis

As stated in Table 9, the molecular dynamics simulation was generated with a 100 ns frame time interval for the purpose of analyzing the binding free energy calculation following MM/GBSA. The findings revealed that, on average, complexes (Compound A, B, C, and D) at nucleotide binding sites had better binding free energies at post simulation period (−97.15, −57.11, −73.07, and −42.02 kcal/mol) compared to pre-MM/GBSA complexes, but the complexes (Compound 2 and 3) at inter-domain binding sites had comparable binding free energies (−45.49 and −45.38 kcal/mol) compared to pre-MM/GBSA free energies. As there is less variation among the pre and post MM/GBSA energies of complexes at the inter-domain site, they can be considered as strong binders (Table 10). It was observed that for the binding of compounds at both binding sites, the MM/GBSA dG bind coulomb, and MM/GBSA dG bind solv GB energies majorly contributed to the binding energies of the complexes.

**TABLE 9:** Post MM/GBSA Binding free energies of lead compounds in complex with nucleotide binding site.

Compound ID	MM/GBSA dG bind (kcal/mol)	MM/GBSA dG bind coulomb (kcal/mol)	MM/GBSA dG bind covalent (kcal/mol)	MM/GBSA dG bind Hbond (kcal/mol)	MM/GBSA dG bind Vdw (kcal/mol)	MM/GBSA dG bind packing (kcal/mol)	MM/GBSA dG bind solv GB (kcal/mol)
Compound A	−97.15	−203.81	3.41	−11.7	−51.54	−4.27	178.83
Compound B	−57.11	−46.22	6.88	−5.08	−45.17	−0.74	48.21
Compound C	−73.07	−181.11	2.97	−13.71	−41.81	−2.8	168.55
Compound D	−42.02	−104.1	10.51	−12.59	−53.24	−3.21	125.13

**TABLE 10:** Post MM/GBSA Binding free energies of lead compounds in complex with inter-domain binding site.

Compound ID	MM/GBSA dG bind (kcal/mol)	MM/GBSA dG bind coulomb (kcal/mol)	MM/GBSA dG bind covalent (kcal/mol)	MM/GBSA dG bind Hbond (kcal/mol)	MM/GBSA dG bind Vdw (kcal/mol)	MM/GBSA dG bind packing (kcal/mol)	MM/GBSA dG bind solv GB (kcal/mol)
Compound 2	−45.49	−288.66	2.55	−1.47	−33.56	−3.71	294.33
Compound 3	−45.38	19.15	2.69	−3.04	−31.74	−1.72	−18.14

## Conclusion

Our research sought to identify a potential treatment for *S. epidermidis* infection and looked at FtsZ inhibitors previously examined among other pathogenic species found in the literature.

Virtual screening methods showed ten molecules as best hits, further evaluated by analyzing their ligand interactions and binding affinity. The shortlisted five compounds based on binding free energy analysis were further taken up for additional *in silico* investigations like ADME/T prediction, molecular dynamics simulations, and post MM/GBSA analysis. Based on all the results obtained, we conclude that, chloro-derivative of GTP (Compound A), naphthalene-1,3-diyl bis(3,4,5-trihydroxybenzoate (Compound B), Guanosine triphosphate (GTP) (Compound C), morpholine derivative of GTP (Compound D), and 1-(((amino(iminio)methyl)amino)methyl)-3-(3-(tert-butyl)phenyl)-6,7-dimethoxyisoquinolin-2-ium (Compound 2), and Chlorogenic acid (Compound 3) can act as potential inhibitors of FtsZ protein interrupting cell division mechanism thereby limiting the growth of *S. epidermidis.* The current study will be very helpful for future research to develop targeted therapeutics to combat infection. To establish their status as novel compounds against *S. epidermidis*, the identified inhibitors will be expanded through experimental research in the future.

## Data Availability

The original contributions presented in the study are included in the article/Supplementary Material, further inquiries can be directed to the corresponding author.
