# Supplementary figures and images for: Homology modeling, virtual screening, molecular docking, and dynamics studies for discovering Staphylococcus epidermidis FtsZ inhibitors
Source: Front Mol Biosci. 2023 Mar 3;10:1087676. doi: 10.3389/fmolb.2023.1087676 (PMC10020519; doi:10.3389/fmolb.2023.1087676)

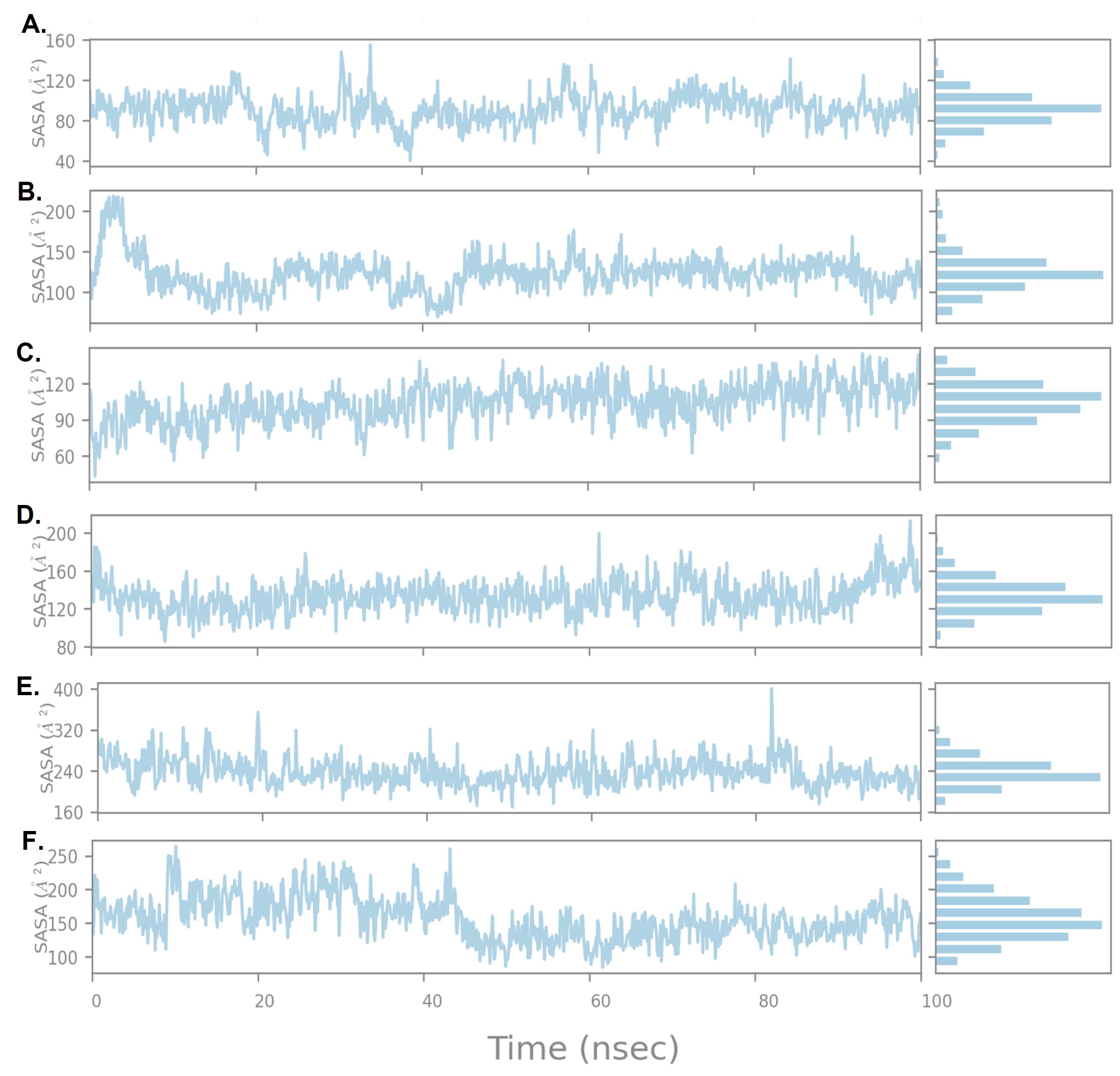

Supplement: Supplementary file 1 [file Image3.jpeg]

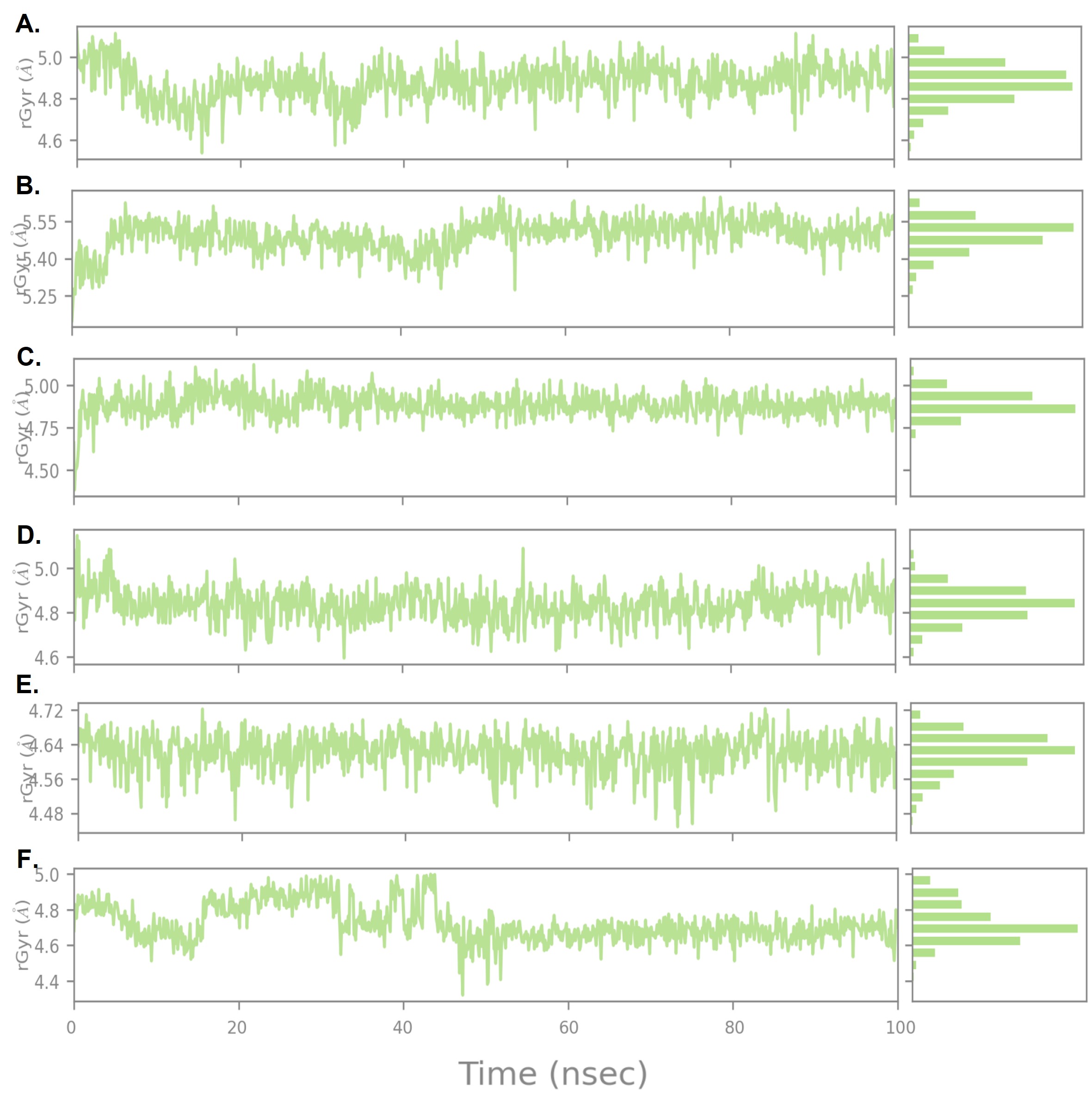

Supplement: Supplementary file 2 [file Image1.jpeg]

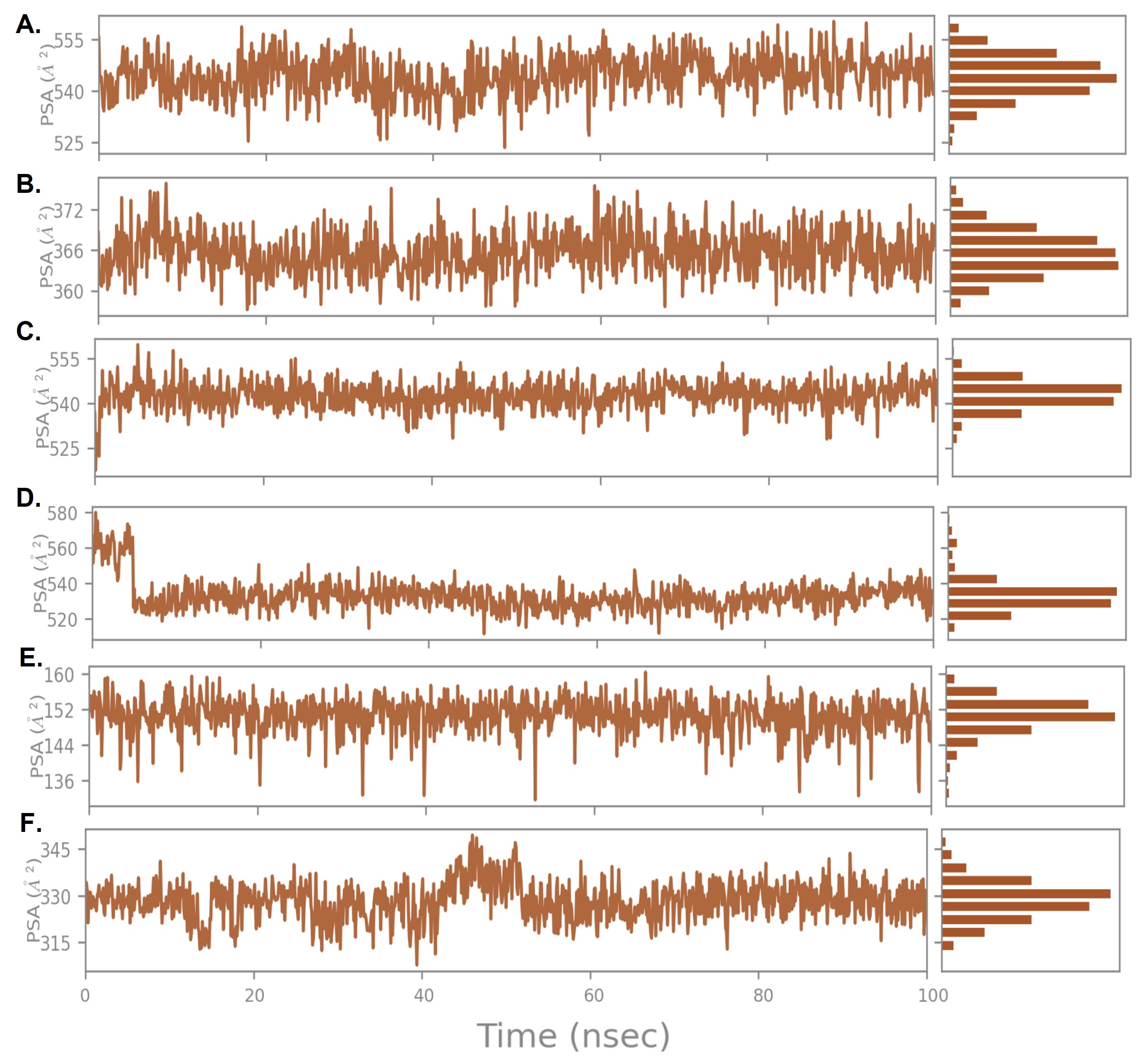

Supplement: Supplementary file 3 [file Image4.jpeg]

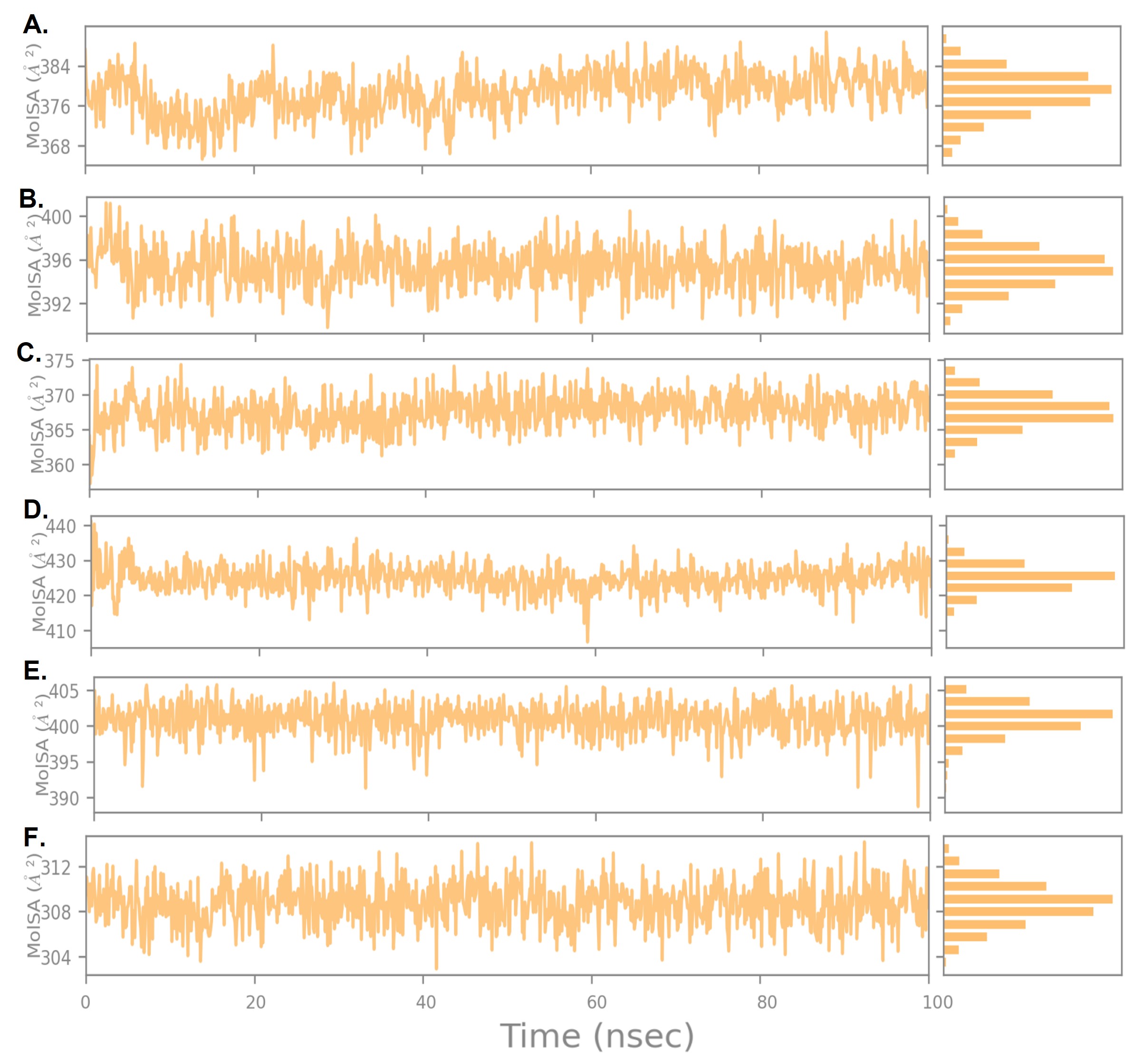

Supplement: Supplementary file 4 [file Image2.jpeg]

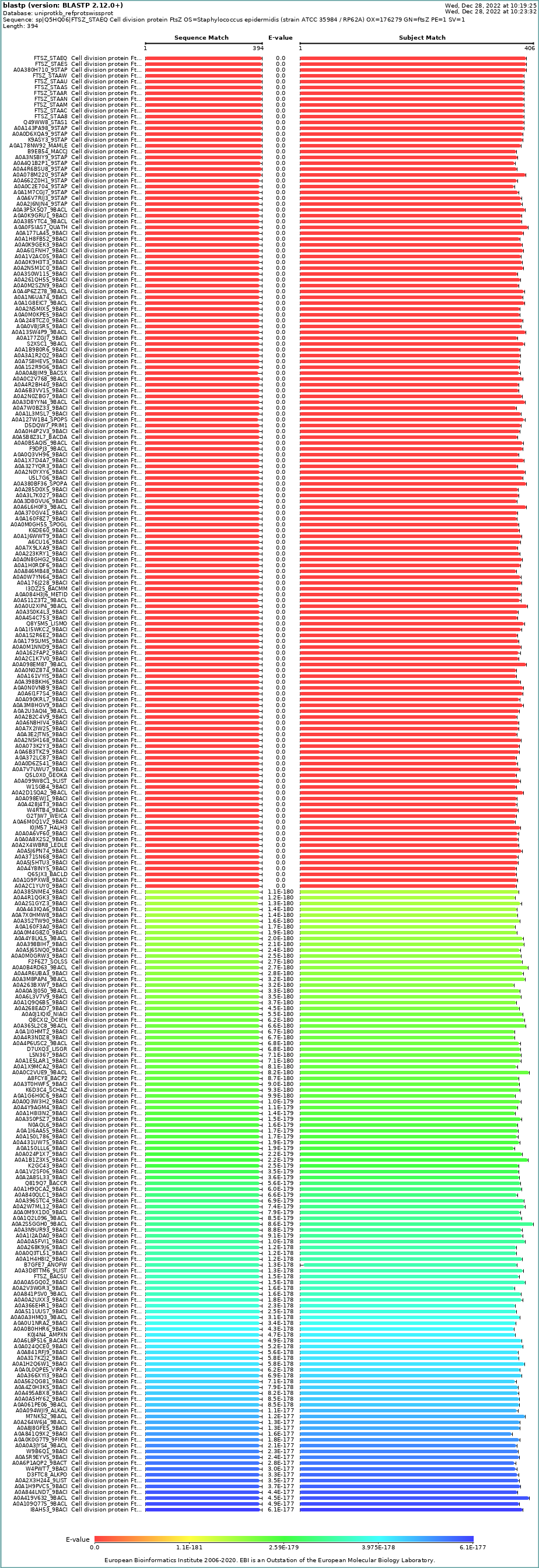

Supplement: Supplementary file 5 [file Image5.png]
